# Analyzing linguistic variation and change using gamification web apps: The case of German-speaking Europe

**DOI:** 10.1371/journal.pone.0225399

**Published:** 2019-12-11

**Authors:** Adrian Leemann, Curdin Derungs, Stephan Elspaß

**Affiliations:** 1 Center for the Study of Language and Society, University of Bern, Bern, Switzerland; 2 Department of Geography, University of Zurich, Zurich, Switzerland; 3 Fachbereich Germanistik, University of Salzburg, Salzburg, Austria; University of California Santa Barbara, UNITED STATES

## Abstract

Research on regional linguistic variation typically involves data collection in the field. This process itself can take up several months if not years. In the present study we demonstrate how we can use web interactives in collaboration with media outlets for a fast gathering of regional, sociolinguistic data. In collaboration with *SPIEGEL ONLINE* and *Tagesanzeiger*, we developed a web interactive that predicts users’ regional backgrounds from within German-speaking Europe. More than 1.9M people have participated in the interactive, more than 770K users have provided metadata. Said metadata allowed us to capture regional variation in language as of today, which we can compare to historical survey data–enabling us to track the evolution of German in Europe over the past 40 years. We report on regional levelling of lexical variants, a process which appears to be particularly prevalent in the northern parts of German-speaking Europe. We further found an effect of (former) national and regional borders on language use. This innovative paradigm allows us to collect sociolinguistic data of an unprecedented scale–at the same time it presents significant challenges, both of which–benefits and challenges–will be discussed in this contribution.

## 1. Introduction

German is one of the world’s major languages and is spoken in many regions across Europe and other parts of the world [1,2]. With 91 million speakers, it is the most widely spoken first language in Europe (ibid.) and the most widespread official language in Germany, Austria, and Switzerland. German has been characterized as the ‘probably most diverse language in Europe’ [3]. The linguistic situation in the German-speaking countries is complex: the spectrum of spoken varieties includes standard varieties on the one hand (e.g. Standard Swiss High German and more than just one standard variety each in Germany and in Austria), and local dialects and supralocal regional varieties on the other hand. In the north of Germany, dialects are situated furthest away from the standard; speakers who still master a Low German dialect switch from dialect to a more supraregional or a standard variety [4,5]. In Austria and in the central and southern parts of Germany, we find a continuum between dialect and standard, where the choice of the register depends on the situation, emotional engagement, and other factors. In Switzerland, the situation is even more different: supralocal regional varieties do not exist, but we find a functional diglossia between standard and dialect–where the standard variety is primarily used in writing, in formal speeches, and in school, whereas dialect is spoken otherwise [6] and even used in informal writing, such as private text messages. One level of language in which these varieties of German are particularly different from one another is the lexicon: for ‘bread roll’, for example, speakers in northern Germany say *Brötchen*, people in Baden-Württemberg call it *Weck(er)le*, in Bavaria and most of Austria *Semme(r)l*, and in German-speaking Switzerland one typically hears *Mütschli* or *Brötli*.

This type of regional variation in the lexicon has been thoroughly studied in the *Wortatlas der deutschen Umgangssprachen (WDU)* [7], i.e. ‘Word Atlas of German Regional Varieties’ (Eichhoff 1977–2000), launched in the early 1970s. Eichhoff sent questionnaires by post mail to local residents in 402 localities from across the Federal Republic of Germany, the German Democratic Republic, Austria, German-speaking Switzerland, and South Tyrol. East Belgium, Luxembourg, and Liechtenstein, where German is an official language, were not sampled in the *WDU*. In about one third of the localities, he and his collaborators were able to interview their informants so that in these cases, it was actually the interviewer, not the interviewees who completed the questionnaire. The questionnaire used a mainly onomasiological approach: the informants were asked to provide the local expression for a certain concept, e.g. the local word for ‘the boy’, for ‘to chat’, or for ‘wallet’. The questionnaire also included images, for example a clock showing the time ‘8:15’, a ‘carrot’, or items in which the informants were asked to translate short phrases or choose between a short list of variants. Unlike traditional dialect surveys, Eichhoff focused on participants from the younger and middle generation in urban areas, and the participants were asked not about their own language use, but to report variants they believed are typically in use in the respective locality. In the case of Germany and Austria he thus targeted more regional, supralocal varieties, whereas in the case of German-speaking Switzerland, he elicited dialects. He surveyed, for example, the regional variants of the Standard German word *der Junge* (‘the boy’). He retrieved the following regional distribution: in the north of Germany, *Junge* was reported most dominantly, while *Bub*, *Bu(e)(b)* or *Bua(b)* were particularly prevalent in central Germany, Switzerland, and Austria. *Knabe* was reported in northern Bavaria and Baden-Württemberg and *Bersch* (/*Bursch*) in the Austrian state of Styria (cf. *WDU* [7] vol. 1, map 1). Maps from this survey were published in four volumes between 1977 and 2000. [8] replicated Eichhoff’s method for the eastern German-speaking part of Belgium, the results were published in a supplementary volume to the *WDU*. Other projects in the same vein, but focusing on certain regions of Germany, include the *WSAH* (‘Word geography of urban colloquial language in Hessia’, published 1988, [9]), the *ALRH* (‘Word atlas on colloquial language in rural Hessia’, published 2010, [10]), and the *WSU* (‘Word atlas of urban colloquial language’ in the eastern states of Germany, originally planned as an atlas on urban colloquial lexis in the German Democratic Republic, but only published in 1997 [11], seven years after its dissolution).

The second survey of central importance to the current study is the *Atlas zur Alltagssprache*, short *AdA* (‘Atlas of everyday language’, [12,13]) by Elspaß and Möller. In the early 2000s, Elspaß & Möller began sending out online questionnaires which–like Eichhoff–aimed at eliciting everyday language. The *AdA* is particularly geared at capturing lexical variation, but also includes questions on morphosyntax, phonetics, and pragmatic features (like routine formulae). One *AdA* survey from 2006 was directed at perceptual data [14]. Initially, Elspaß & Möller targeted localities’ participants from the original 402 original *WDU* localities. This first survey attracted 1,763 participants, and it was followed by new surveys at regular intervals. Over the years, the number of participants snowballed so that in the tenth survey over 20,000 people provided data. The *AdA* is still ongoing and is now in its 12^th^ survey round.

It is reasonable to assume that the distribution of lexical variants in space has changed since the time of the *WDU*, the data of which was mainly collected in the 1970s –i.e. c. 40–50 years ago. This is plausible because (a) the lexicon is typically more prone to change compared to phonetic / phonological domains [15–17] and (b) because *WDU* specifically asked for variants of a form of language which is spoken everyday–thus more likely (but not necessarily) non-standard–and which is regionally-colored, i.e. a form of language which is less stable and more in flux than traditional local dialects on the one end or standard varieties on the other end [18].

The hypothesis that the regional distribution of these variants has changed over the past decades was tested by [19] using a change in real-time framework [20]. He compared the regional distribution of eleven *WDU* maps (nine lexical variables, two syntactic variables) from the first two volumes of the *WDU*, published in 1977 and 1978, to eleven equivalent maps from an online survey conducted in 2002 –thus studying language change across a time span of c. 25–30 years. The online survey, a pilot study for the subsequent *AdA*, provided data from roughly 1,500 respondents across Germany, Austria, German-speaking Switzerland and northern Italy. [19] reports three main trends that emerged from the study: (i) On the one hand, there are variants that show tendencies of diatopic convergence, i.e. a levelling of lexical variants–where small-scale variants are receding while large-scale, more supralocal variants are spreading. This pattern applied to five of the eleven cases and to variants used in Germany only. (ii) On the other hand, but to a lesser degree, there is a trend of what he refers to as ‘divergence’, i.e. the effect of national and political borders: in two notable instances (regional forms for the temporal concepts ‘Saturday’ and ‘5:45’), variants appear to follow political borders relatively closely–or, more precisely, what *used* to be the political border between West and East Germany, even thirteen years after the effective fall of the Iron Curtain. On a side note: the *WDU* maps based on data from the 1970s displayed only first signs of the divergence process. German-speaking Switzerland and to some extent also Austria often maintain their own variants, with isoglosses typically following along the border to Germany. (iii) Thirdly, in two other instances (the word for ‘carrot’ and the modal particles *halt* and *eben*), formerly southern variants (*Karotte* and *halt*) had spread to the northern parts of Germany. This is all the more striking as the general trend in other linguistic domains, notably in the phonetic-phonological domain, is that northern German forms have spread to southern Germany; [21] claimed that northern German variants were more prestigious than southern variants. In two other instances the overall pattern hardly changed; both of them were cases of grammatical variation (diminutive suffixes and the use of the auxiliaries *sein* ‘to be’ and *haben* ‘to have’ in analytical forms of the perfect tense of the position verb *sitzen* ‘to sit’).

In the present contribution we follow suit with the line of research conducted by [19]–albeit with substantially more data and more variables. Like [12], we also used an online tool to crowdsource data. However, we did not opt for a traditional survey interface, but rather for a new paradigm of collecting data via a web interactive. Over the past couple of years, Leemann et al. began developing mobile and web-apps which enable crowdsourcing of regional language data from speakers through a quiz [16,22]. The users go through this quiz on regional language use and, at the end of the quiz, the app tells them where they are from, based on their linguistic features. In the quiz for German-speaking Europe, which this paper is about, we asked users, for example, what their local word is for ‘non-professional soccer playing’ or for ‘(second) breakfast that people have at their workplace’, thus adopting the onomasiological approach employed for the *WDU* and the *AdA*. After 24 such questions, the web interactive tells the individual participant, depending on which variants they indicated, where they are from within German-speaking Europe. The user can then evaluate the quiz result, provide metadata on age and gender, and submit this data. This enables us to collect regional language data on a large scale, which we can subsequently use to compare to historical data, in this case Eichhoff’s data, thus enabling the study of linguistic change (for more details see section 2 Methods). The web interactive that collected the data which the present study is based on was developed by Adrian Leemann, Marie-José Kolly, Timo Grossenbacher, Marc Brupbacher, Daniel Wanitsch, and colleagues from the online edition of the news magazine *SPIEGEL ONLINE* and the Zurich-based newspaper *Tagesanzeiger* [23].

Going into the study, we have the following prediction:

We expect substantial lexical change to have occurred since the 1970s, the time Eichhoff’s survey was conducted. We will examine this by performing analyses of real-time change (i.e. comparing historical *WDU* data from the 1970s to contemporary data we crowdsourced in 2015) and analyses of apparent-time change (i.e. comparing the spatial distributions of different age groups in our contemporary data). If the older generation speakers in our apparent analyses show less change when compared to *WDU* than the younger generation does, we can be fairly certain, that actual language change has taken place. We suspect that both analyses will point in a very similar direction, as crowdsourcing methodology has been shown to indicate very similar diffusion patterns, similar to those found in more traditional paradigms [16].

The article is structured as follows: section 2 will present the methods to study the above prediction. Given that Eichhoff’s *WDU* plays such a central role as a benchmark for our analyses of linguistic change, we will also highlight Eichhoff’s methodological procedures in the methods section where appropriate. In Section 3 we will present the main findings, followed by a discussion thereof in Section 4. A large part of the Discussion Section will also be devoted to a critical reflection on the limitations and benefits of this new paradigm of data collection.

## 2. Methods

### 2.1 Web interactive: *Grüezi*, *Moin*, *Servus*

**Fig 1:**
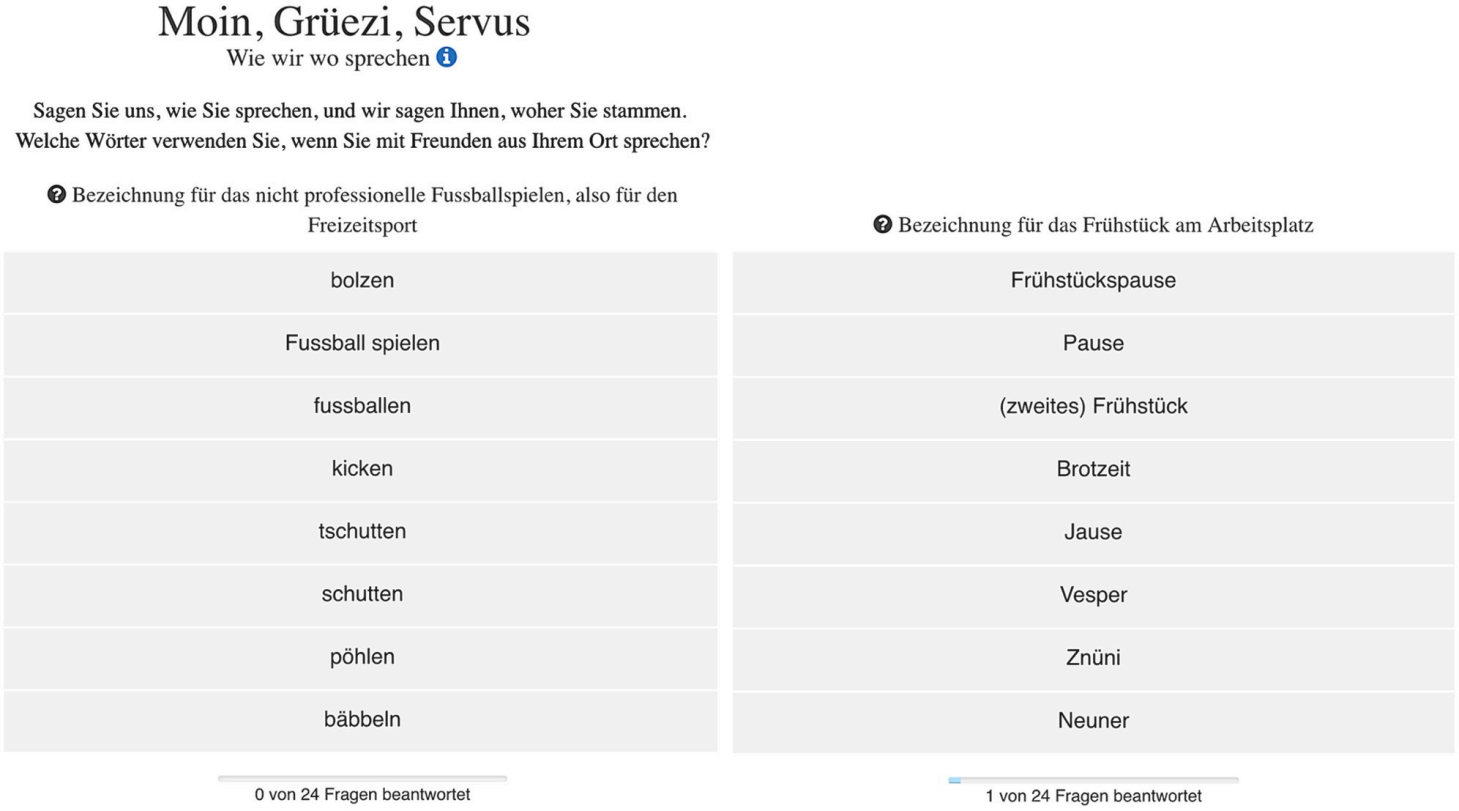
UI showing regional variants for ‘non-professional soccer playing’ (left) and ‘(second) breakfast that people have at their workplace’ (right).

The process of collecting the data for the current study is based on a quiz we developed with *SPIEGEL ONLINE* and Zurich-based *Tagesanzeiger* [23]. Analogous to smartphone apps we had previously developed [16], we developed a web interactive–called *Grüezi*, *Moin*, *Servus*–that prompts users to click on their regional variant for 24 variables. One of the questions was, for example, to indicate their regional word for ‘non-professional soccer playing’–do they say *bolzen*, *Fussball spielen*, *fussballen*, *kicken*, *tschutten*, *schutten*, *pöhlen*, or *bäbbeln*? Or we asked users, what word they use for the ‘(second) breakfast that people have at their workplace’–i.e. *Frühstückspause*, *Pause*, *Frühstück*, *Brotzeit*, *Jause*, or *Vesper*. This user interface (UI) is shown in Fig 1, examples for ‘non-professional soccer playing’ and ‘(second) breakfast that people have at their workplace’ are shown on the left and on the right respectively.

To trigger the user’s linguistic intuition, we asked them to click on the word that they would typically use if they spoke to friends at their location. Once the user had completed the quiz, the web interactive prompted them to indicate metadata on age and gender. Finally, the web interface showed the result of the quiz: a heatmap and the top five localities as pins on a map. The ‘hotter’ (i.e. the redder) the area, the more likely it is that the user speaks like someone from that region–the ‘colder’ (i.e. the bluer), the less likely it is that they are from that region. Once the result has been presented, we then asked users to ‘validate’ this prediction. We asked them to rate the result on a scale from one (very inaccurate) to seven (very accurate). Further, we asked the users to click the point on the map where they believe they, i.e. the people from this locality with the pin, spoke most like they do. Once the user has evaluated the result, they click on ‘submit’. This geotagged data is sent to our servers along with the 24 variants the users selected and metadata on their age and gender.

None of these pieces of information individually or in combination allow for identification of users in the database. Users always had the opportunity to opt out of providing us with their metadata on age, gender, regional variants, and place of origin. This procedure of collecting anonymous user data conforms to the regulations of the Zurich cantonal ethics committee (http://www.kek.zh.ch/internet/gesundheitsdirektion/kek/de/home.html) and the accompanying federal laws on experimentation on humans in Switzerland (http://www.admin.ch/opc/de/classified-compilation/20061313/index.html). We therefore did not seek further ethical approval from cantonal or federal institutional bodies. This new, crowdsourced data can then be compared to the maps provided by Eichhoff, which allows us to track change in language over time.

This method of data collection and user engagement is not new. In the early 2010s, [24] developed a dialect prediction app just for German-speaking Switzerland. Half a year after the release, [25] developed a dialect predictor for American English. Here, participants were asked, for example, which regional word they use for ‘sweetened carbonated beverage’–*coke*, *pop*, *soda*, or other (see also [26]). After 25 such questions, the web interactive tells the American user, which region they are from. This web interactive was largely based on [27]’s Harvard Dialect Survey. Both apps were highly successful: the American web interactive was the most visited content on the *New York Times* site in 2013 [28]. The Swiss app was the most downloaded app in the *iOS App Store* for a number of weeks [29].

When comparing the data from the web interactive with Eichhoff’s data, it has to be remembered that Eichhoff used paper questionnaires and asked one to two informants per locality on their notion of the local word or expression for a certain concept. To account for local variation, the informants could provide more than one variant, but if they did so they were requested to highlight the more frequent of the two (or more) variants. In contrast, the web interactive asked for the user’s own language use and allowed for one variant per variable only. The different methods of data elicitation may put a certain caveat on the comparability of the data sets. However, both methods aimed primarily at the representation of language use at particular localities. In Eichhoff’s data, this was achieved by the informants assuming the role of experts of the local colloquial language, whereas the web interactive collected data per locality to arrive at one (two or three) variant(s) which are inter-individually used at this locality.

### 2.2 Variables

To develop the prediction, we needed a set of maps (i.e. variables) that allowed for a geographical partitioning of German-speaking Europe. We chose variables with different geographical distributions so that very small areas could be distinguished from each other on the basis of unique answer combinations. The variables (and variants) were selected from *AdA*, rather than *WDU* because we wanted to ensure that prediction accuracy was relatively high–which may have been problematic had we used historical data, such as *WDU*, for the prediction. Table 1 shows the 24 variables we selected for the prediction, cf. Table 1.

**Table 1:** Variables chosen for the prediction of the users’ regional background.

Variable	Example variants	# variants	Type
Non-professional soccer playing	*bolzen*, *Fussball spielen*	8	Lexical
Second breakfast at work	*Frühstückspause*, *Neuner*	8	Lexical
Red peppers	*die Paprika*, *der Peperoni*	5	Lexical
Pancakes	*Pfannkuche(n)*, *Eierkuchen*	12	Lexical
Breadman	*Grittibänz*, *Klausenmann*	13	Lexical
Beef patty	*Bulette*, *Hackloable*	16	Lexical
to chat	*quatschen*, *plaudern*	10	Lexical
Slippers	*Latschen*, *Pantoffeln*	10	Lexical
Time 10:15	*Viertel nach zehn*, *viertel elf*	4	Lexical
Happy New Year!	*Frohes Neues*, *Prosit Neujahr*	10	Lexical
Slingshot	*Steinschleuder*, *Zwille*	11	Lexical
Armchair	*Sessel*, *Fauteuil*	3	Lexical
School exam	*Probe*, *Schulaufgabe*	10	Lexical
Hiccups	*Schluckauf*, *Hicks*	25	Lexical
Drawing pin	*Reissnagel*, *Heftzwecke*	6	Lexical
Wallet	*Portemonnaie*, *Geldbeutel*	6	Lexical
Dull	*Langweilig*, *fad(e)*	2	Lexical
Mashed potato	*Kartoffelbrei*, *Püree*	14	Lexical
3rd p. pl. *haben wir*	*ham*, *hei*, *hum*	15	Morphological
Pronunciation of ‘15’	*fuffzehn*, *fuffzeh*	4	Phonetic
Pencil case	*Federmappe*, *Federtasche*	7	Lexical
Demonstrative *das*	*das*, *des*, *dis*, *dos*	10	Phonetic
Heel end	*Kanten*, *Raftl*, *Kruste*	53	Lexical
10 cent coin	*Groschen*, *Zehnerla*	9	Lexical

The phonetic variable ‘Pronunciation of ‘15”, for instance, featured four variants in *AdA*: *fuffzehn*, *fuffzeh*, *fünfzehn/füfzäh*, and *fuchzehn*. The vast majority of variables in the quiz are lexical in nature (21/24), only two were phonetic variables, and only one (3rd p. pl. *haben wir*) can be categorized as morphological. An overlay of these 24 maps revealed a fine-grained regional partitioning of German-speaking Europe.

Using this material, the web interactive is able to estimate the user’s regional origin: underlying the prediction is a table which has one row for each locality and one column for each regional variant. The algorithm aggregates all the hits (i.e. when a locality features a specific variant) and identifies the locality with the most hits. As a result, the user is presented with the top five from a total of 487 *AdA* localities (cf. [22,30] for a more detailed description of the prediction algorithm used in the apps developed).

### 2.3 Participants

**Fig 2:**
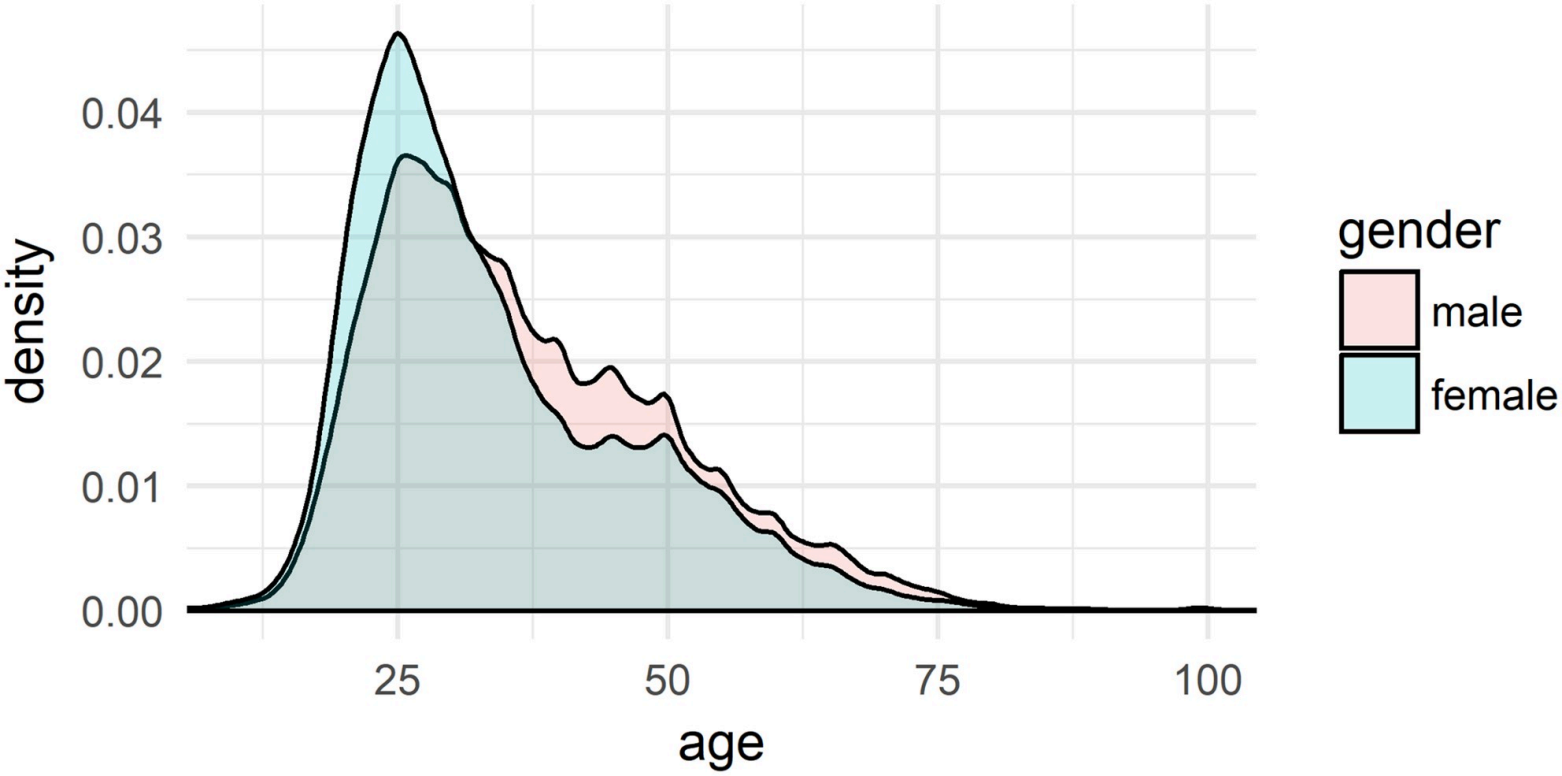
Density distribution of users by age and gender.

The quiz garnered widespread participation, particularly so in the first days following its release on 25 April 2015. Until today, more than 1.91 million users participated, of which 46% were females, 54% were males. The largest segments of participants come from the age bracket 25–34, with a share of 33.5% of all participants. This is roughly the age bracket which Eichhoff aimed at in the survey for *WDU*. Participants 65+ only made up 5.5%. Of these 1.91M participants, 774,524 evaluated the result and submitted their metadata (cf. 2.1). Fig 2 shows the density plot for these 774,524 participants by age and gender.

In Fig 2, the full area below the density line equals 1, i.e. 100% of the participants. As is evident from the density distribution shown in Fig 2, our data shows a substantial sampling bias towards participants between c. 18 and 45 years of age, while users <18 and >45 are heavily under-sampled. Gender is also not sampled representatively: women between 20 and 28 are sampled more substantially, while men are sampled more than women for the age bracket of 35 to 75. Interestingly, though, the sampling obtained aligns well with the one in Eichhoff’s studies, who was aiming for the ‘younger’ generation [7]. Eichhoff was targeting two representative speakers for each locality. The parents of these two speakers, too, should have come from this locality. Often, however, he was able to collect data only from one person per locality. Eichhoff does not specify gender distribution in his sampling. From the individual metadata it appears that there were twice as many male participants than females.

### 2.4 Localities

**Fig 3:**
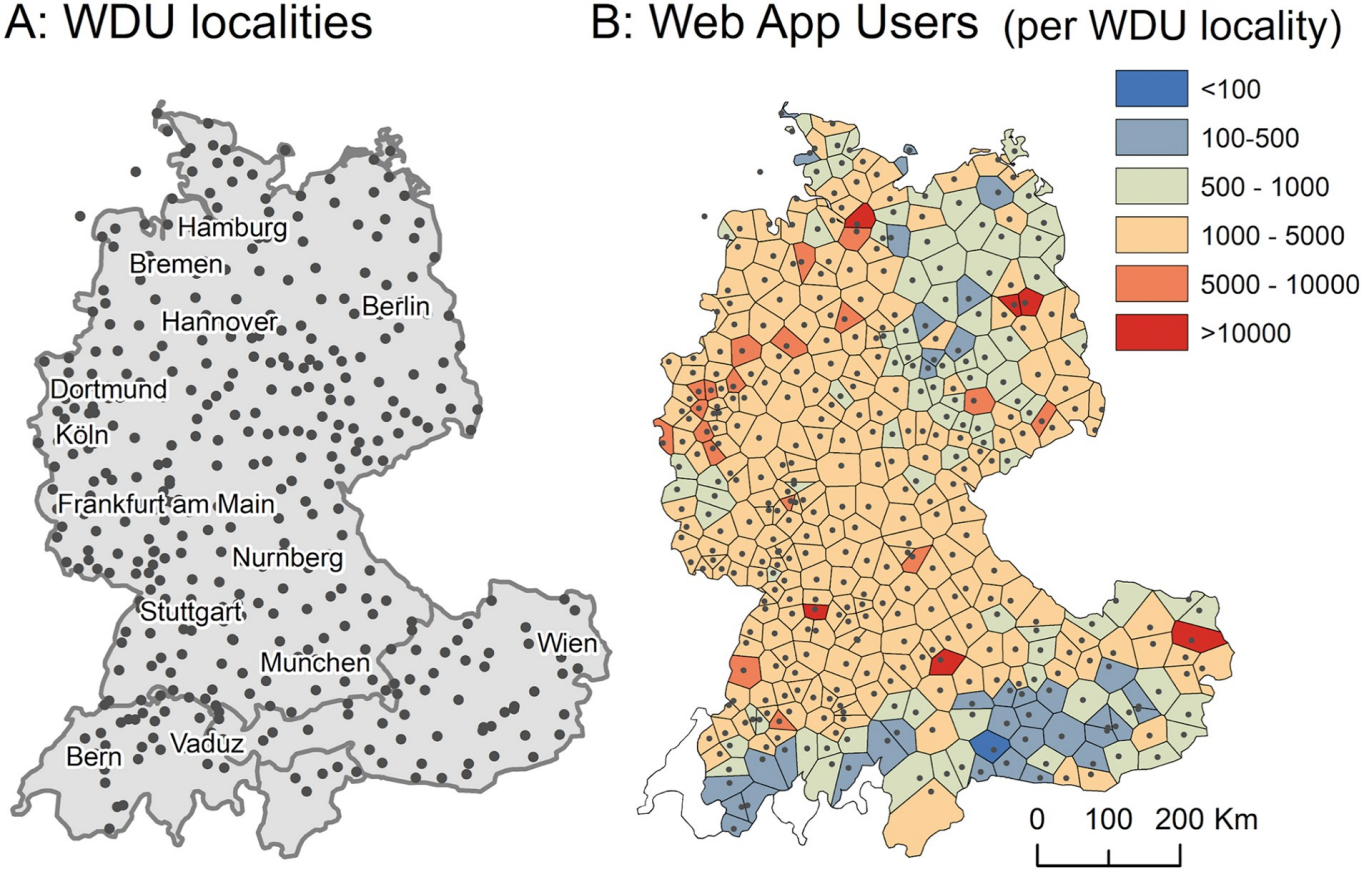
Distribution of localities in the historic *WDU* survey (A) and contemporary data (B). Counts of localities in the contemporary data are aggregated for each *WDU* locality.

While Eichhoff sent questionnaires to residents in 402 localities across German-speaking Europe, our web interactive sampled users from more than 22,000 localities. Fig 3 shows the distribution of localities in *WDU* on the left, and localities that were crowdsourced in our contemporary data (right)–the latter shows counts of localities in the current data that were aggregated for each locality in *WDU* cf. Fig 3.

Our users clicked on 22,828 unique localities (see 2.1), which we retrieved via Google reverse geocoding API. Fig 3 shows these as Thiessen polygons, aggregated for each locality in *WDU*. The Thiessen polygons often comprise urban regions, such as around Vienna, Berlin or Zurich. For most localities our data features 100–1000 participants. More urban regions and regions with major city hubs typically feature between 1000 and >5000 participants. As an example, cities like Zurich, Berlin, or Vienna along with their urban regions show the following areal (as represented by the respective polygon) and user distributions: Zurich (1,690km^2): 6,659, Berlin (890km^2): 22,711, and Vienna (460km^2): 11,758.

### 2.5 Processing of historical material

For the present study, we compared 14 variables from our contemporary data (2015) to those found in *WDU*. The other ten variables were not comparable as they were not elicited in *WDU* but only in *AdA*. These 14 variables include: ‘(second) breakfast that people have at their workplace’, ‘non-professional soccer playing’, ‘pancakes’, ‘breadman’, ‘beef patty’, ‘chatting’, ‘slippers’, ‘10:45’, ‘slingshot’, ‘hiccups’, ‘wallet’, ‘heel end’, ‘pencil case’, and ‘mashed potatoes’. This comparison allows us to perform analyses of language change in real and apparent time [20]. Depending on when the *WDU* volumes were published, we performed analyses of real-time change between 23 and 44 years. For us to perform the comparison, the original 14 *WDU* maps–printed on paper–were scanned and georeferenced, using standard GIS software, and manually converted into vector format. All digitized maps are available as shapefiles from a GitHub repository [31].

### 2.6 Comparison to historical material

**Fig 4:**
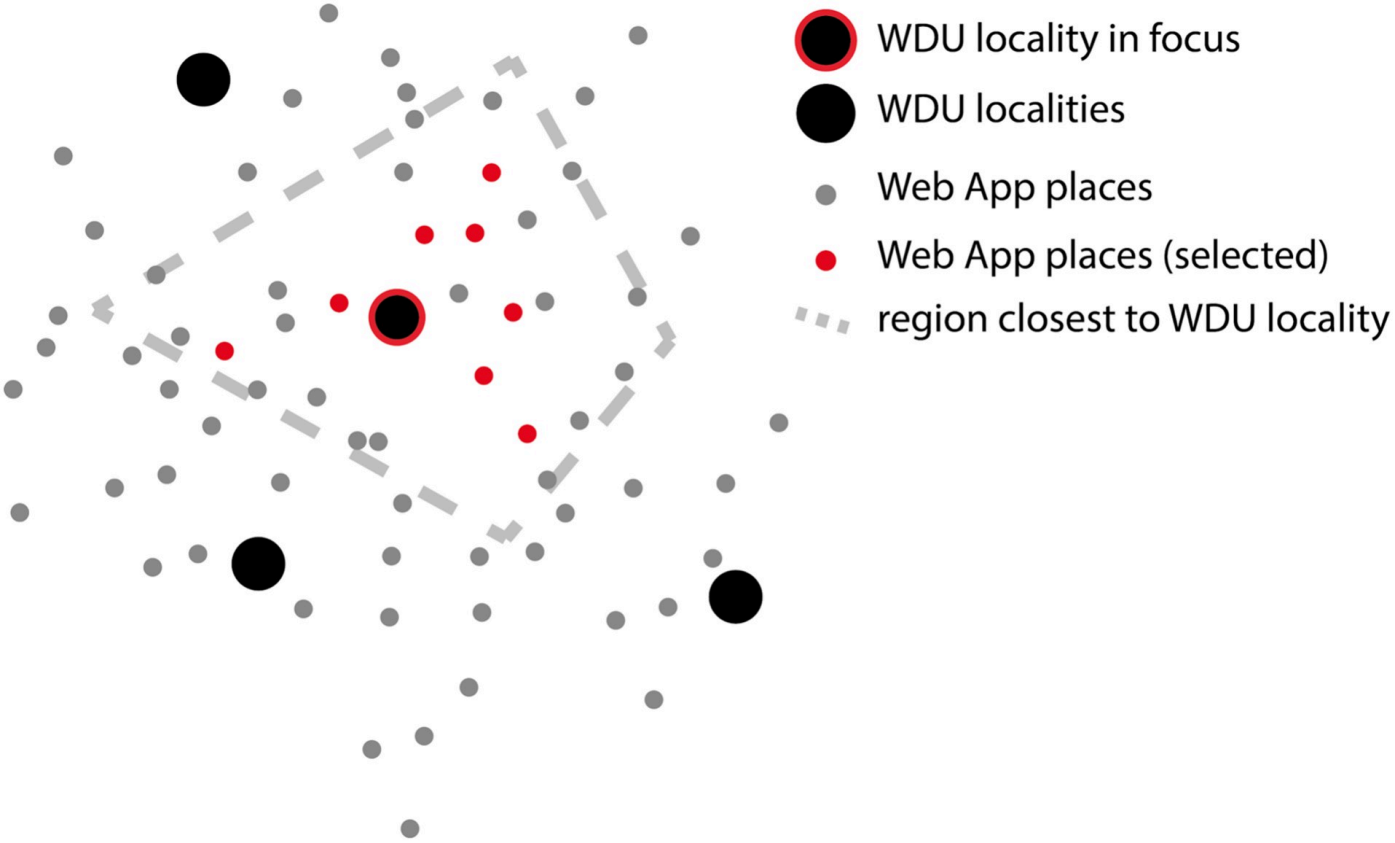
Sketch for computing the degree of change for each *WDU* locality.

The comparison of the current data, collected through the web application, with the historical *WDU* maps is aggravated by the different spatial granularity of the two datasets, i.e. 22,828 localities vs. 402 *WDU* localities. The comparison results in a measure of degree of change between the historical and the current data. The degree of change is computed for each of the 402 *WDU* localities and for each variable individually. The methodology for computing the degree of change for one *WDU* locality is sketched in Fig 4 and includes the following steps:

1. For each *WDU* locality in question (black dot with red outline), the area which is closest to the respective locality–relative to the neighboring *WDU* localities–is identified, using voronoi tessellation (grey dashed line), i.e. without creating overlaps or gaps between the localities. Voronoi polygons for all *WDU* localities are shown in Fig 3, right panel.
2. Within this area, *N* localities from the web app (grey dots) are drawn at random, with localities closer to the *WDU* locality in question having a higher probability to be selected. These are called *selected localities* (red dots). This step of selecting *N* places is explained in further detail below.
3. The linguistic information aggregated over all selected localities (red dots) is compared to the single linguistic information available for the *WDU* locality in question (black dot with red outline). Step 3 is not covered in Fig 4.

The aggregated information from the selected *N* Web App places results in a frequency of use for each variant, for each linguistic variable. This frequency distribution enables the computation of the degree of change measure. If the variant used at the *WDU* locality in question is not used at any of the *N* selected places from the web app, change for the respective variable is 100%. Accordingly, if all *N* selected places use the same variant as used at the *WDU* locality in question, change is 0%. Degree of change is thus identical to the conditional probability of observing a variant at the *WDU* locality in question, given the distribution in the selected, and proximate, web app localities. In the infrequent case where *WDU* indicates two variants in use for one locality, both historical variants are compared to the contemporary data; yet our maps then only show the change that was smaller. The same methodology as described above is used to compute degree of change for different age groups (see section 3.2), the only difference being that prior to the first step, the web app data is filtered, such that only information from the respective age group is retained and used in the computation.

A few more details concerning step (2): the number of selected web app localities *N* is chosen to be 100, if 100 or more places are available for comparison. The rationale for considering ‘only’ 100 randomly selected, nearby localities is twofold. One, subsampling leads to manageable computing times. Two–and probably more importantly–subsampling warrants that the unbalanced distribution of web app localities (cf. Fig 3, right panel) does not impact the computation of degree of change values, such that some localities show a higher degree of change simply because more and richer input information is available. In the case where less than 20 web app localities are available for step (2), we consider a calculation of degree of change not meaningful–and thus no degree of change is computed. Fig 3 (right panel) reveals that only one *WDU* locality exhibits less than 100 nearby web app localities, namely 35 (shown as a dark blue polygon in western Austria). Hence, this threshold value of *N* ≥ 20 localities only comes into effect for the computations of degree of change for different age groups (cf. section 3.2), which requires additional stratification of the input data and thus leads to smaller sample sizes. Note that we can only draw comparisons to the original *WDU* localities, which, unfortunately, do not include datapoints in East Belgium, Luxembourg, and Liechtenstein (cf. Introduction). The data underlying this study can be retrieved from here: https://osf.io/49tcj/.

## 3. Results

Following up on the prediction stated in section 1, we show findings of the current study in three domains: (i) change in real-time–i.e. comparing our contemporary data to the historical data provided in [7] and (ii) change in apparent-time–i.e. comparing different age groups between each other in our contemporary dataset only, as a means of ‘validating’ the trends found in (i). Section (i) will be the main focus of our findings as three broad patterns appear to emerge from our data: (a) regional levelling (convergence), i.e. what have been dominant variants in the past appear to have become even more dominant today and, as a result, push aside variants with localized distributions only; (b) the north and center of German-speaking Europe appear to have changed more than southern parts (e.g. southern Germany, Austria, and especially German-speaking Switzerland); (c) substantial change in Eastern Germany. The reporting of results was thus structured accordingly; we present findings of variables that show representative trends of the 14-variable set a whole.

### 3.1 Change in real-time

#### 3.1.1 Regional levelling

**Fig 5:**
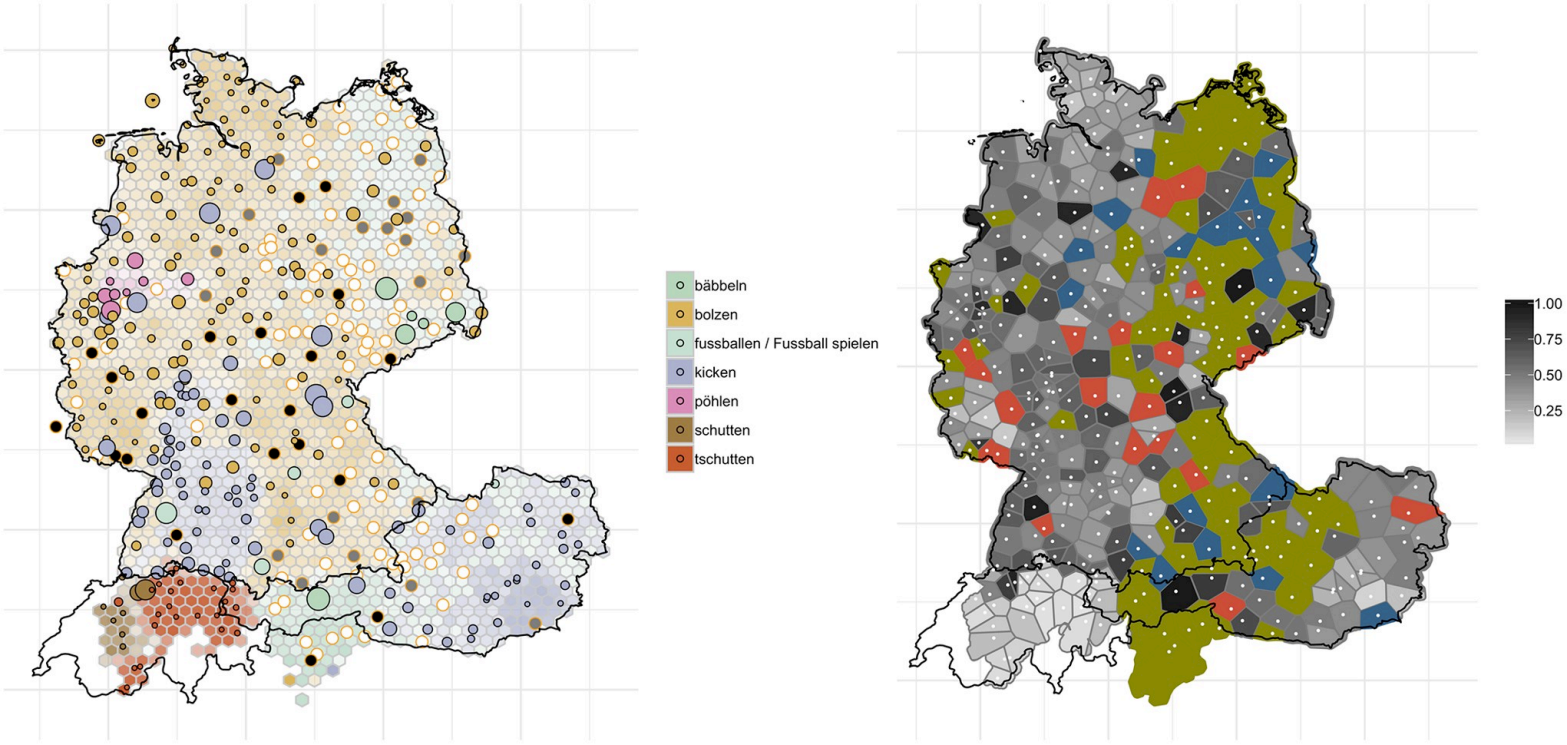
Variant and degree of change maps for ‘non-professional soccer playing’. The left-hand panel shows contemporary data as hexagons with historical data superimposed as dots. The right-hand panel indicates change (black: substantial change, bright grey: very little change).

Fig 5 shows the distribution for ‘non-professional soccer playing’ and how it has changed over the past decades when compared to the *WDU*. The maps can be read as follows: the left-hand map shows the contemporary and historical distribution of variants. Contemporary data is aggregated into 1000 hexagons. We only show the mode, i.e. the most frequent response for each hexagon. The more dominant the variant, the more intense the color of the hexagon. On top of these hexagons we placed the localities from Eichhoff, i.e. the historical data–shown as dots. If the dots are small and, consequently, show the same color as the hexagons in the backdrop, no change has taken place. If the dots are large and show a different color, a change has taken place. The larger the dot, the less we find this historical variant in this locality in the contemporary data. Orange outlines indicate different types of missing data. Orange outlines with black filling represent localities for which there were no responses in the *WDU*. Orange outlines with grey filling highlight rare and particular variants which were not included in the study. Orange outlines with white filling means that users or *WDU* participants did not have a word for the object in question. On the right hand-side we show a map illustrating the degree of change (for computation thereof see section 2.6). Using Thiessen polygons, we demonstrate where and to what degree change has taken place–comparing contemporary data to historical data–light grey means <20% of the nearby selected localities in the web app exhibit a different dominant variant from [7]; black (e.g. >80%) suggests that more than 80% of the localities have a different variant from the one in *WDU*. Red indicates that there are not enough responses (i.e. <20) for that polygon; blue is used for polygons for which the historic Atlas used another variant that we did not query in the quiz; and green is used for polygons where participants from the historic Atlas did not have a word for the item in question. In all these cases–red, blue, and green–we cannot calculate a comparison score.

The trend we observe for the variable ‘non-professional soccer playing’ is one of regional levelling–i.e. the reduction of variants, particularly marked, minority variants, because already widespread forms are becoming even more dominant [32,33]. Regional levelling is particularly evident in the north of Germany, and Germany more generally. To give an example, relatively localized forms such as *bäbbeln* (green on the left panel)–typical for the region of Saxony–or *pöhlen* (pink) in Westphalia appear to have lost ground due to the more generic *bolzen* (light brown)–a trend which is also observable on the corresponding *AdA* map from 2006/2007 (round 4, question 1a). A comparability of *WDU* and *AdA* is clearly possible, since *AdA* has adopted the basic concept of *WDU*, i.e. collecting data at a given locality by asking informants not about their personal language use but the language use which is ‘typical’ for the everyday language at the locality. See Fig 5.

The opposite, i.e. little or hardly any change in lexical variants, is evident for much of the southern regions of German-speaking Europe, in particular German-speaking Switzerland, but also in the eastern part of Austria. In the latter case, *kicken* (dark purple) is still the most dominant form, and in German-speaking Switzerland, *tschutten* (red) and *schutten* (dark brown)–derived from English ‘to shoot’, cf. [34]–are still very much in use. These patterns of change are also evident in the right-hand map shown in Fig 5: some amount of black areas–areas with high degree of change–can be found throughout Germany. German-speaking Switzerland, in particular remains largely unaffected by change in this variable–as indicated by the bright grey polygons in this region. Most of eastern Germany, South Tyrol, but also Upper Austria are green or blue, meaning that (a) a comparison to historical material was not possible as *WDU* respondents reported to not have a variant for the object in question and (b) *WDU* used a variant that was not used in our quiz.

**Fig 6:**
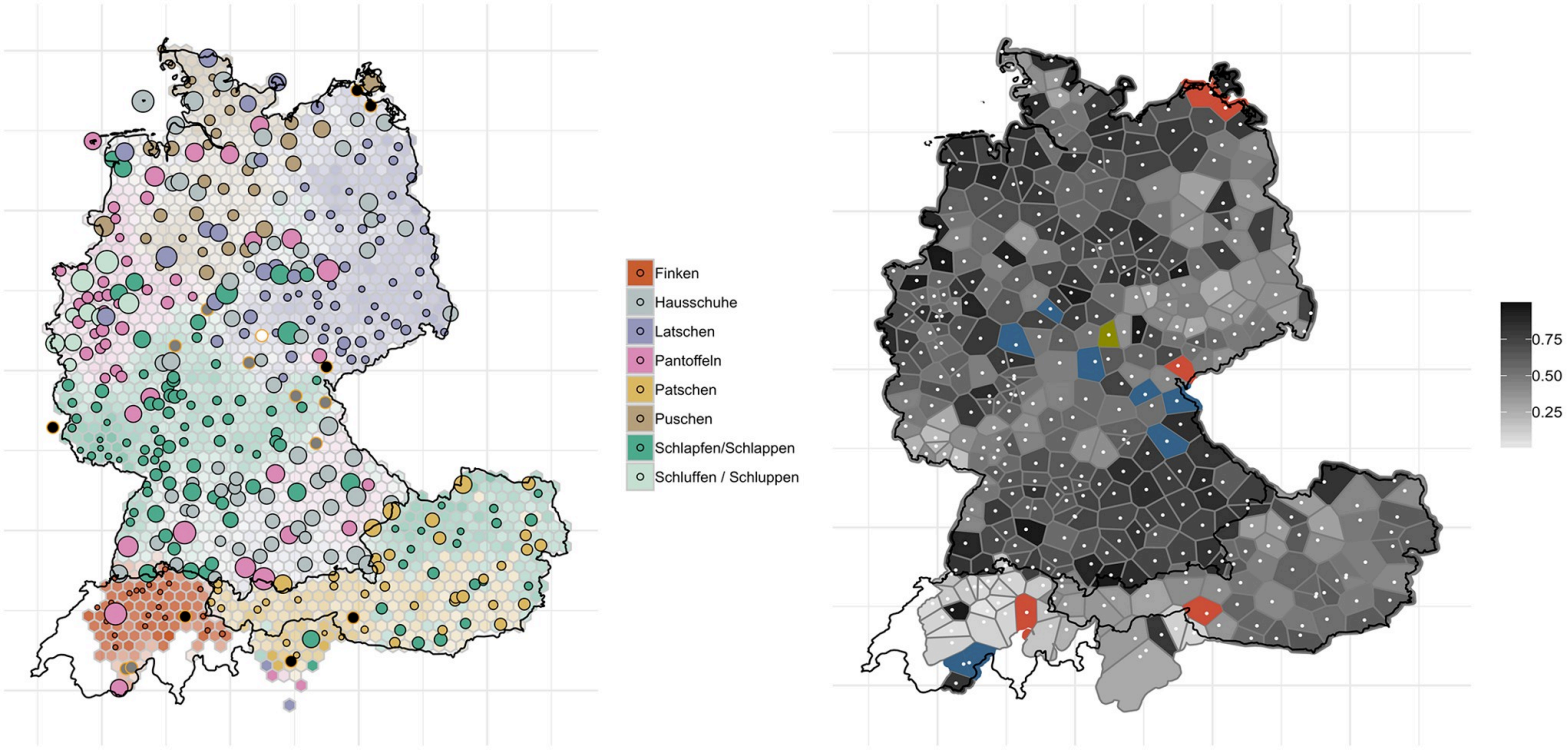
Variant and degree of change maps for ‘slippers’.

We find somewhat similar patterns of levelling and change when looking at the regional variants for ‘slippers’, see Fig 6.

Fig 6 shows the regional distribution for variants of *slippers*. As can be seen in the two maps shown here, substantial change appears to have occurred over the past 40 years. In the west of Germany, for example, the now dominant form *Pantoffeln* (pink) appears to have gained ground at the expense of *Schluppen* / *Schluffen* (light green). Similar patterns of supralocal forms emerging are found in Bavaria, where our current data shows predominantly *Pantoffeln* (pink)–while the historic data also showed a more regional variant including *Hausschuhe* (grey) and *Schlapfen* (dark green). In short–forms which had already been dominant have become even more widespread and have pushed aside more small-scale variants. The right-hand map in Fig 6 suggests that in German-speaking Switzerland and also in South Tyrol, in Austria, and in (north-)eastern Germany, the change found is much less dramatic than in the north, the west or the southeast of Germany. Interestingly, much of the change found for this variable follows the German border in the south: north of German-speaking Switzerland, Vorarlberg, Tyrol, Salzburg, and Upper Austria, substantial change is visible; south of this border, however, regions remained largely unaffected by the change, cf. Swiss German *Finken* (orange-red, left panel), *Patschen* (light brown) in Vorarlberg, Tyrol, Salzburg and Styria and *Schlapfen* in Upper Austria, Lower Austria and Vienna (dark green). This stability in the south is already evident when looking at 2009/2010 data from the *AdA* for this variable (round 7, question 9a).

**Fig 7:**
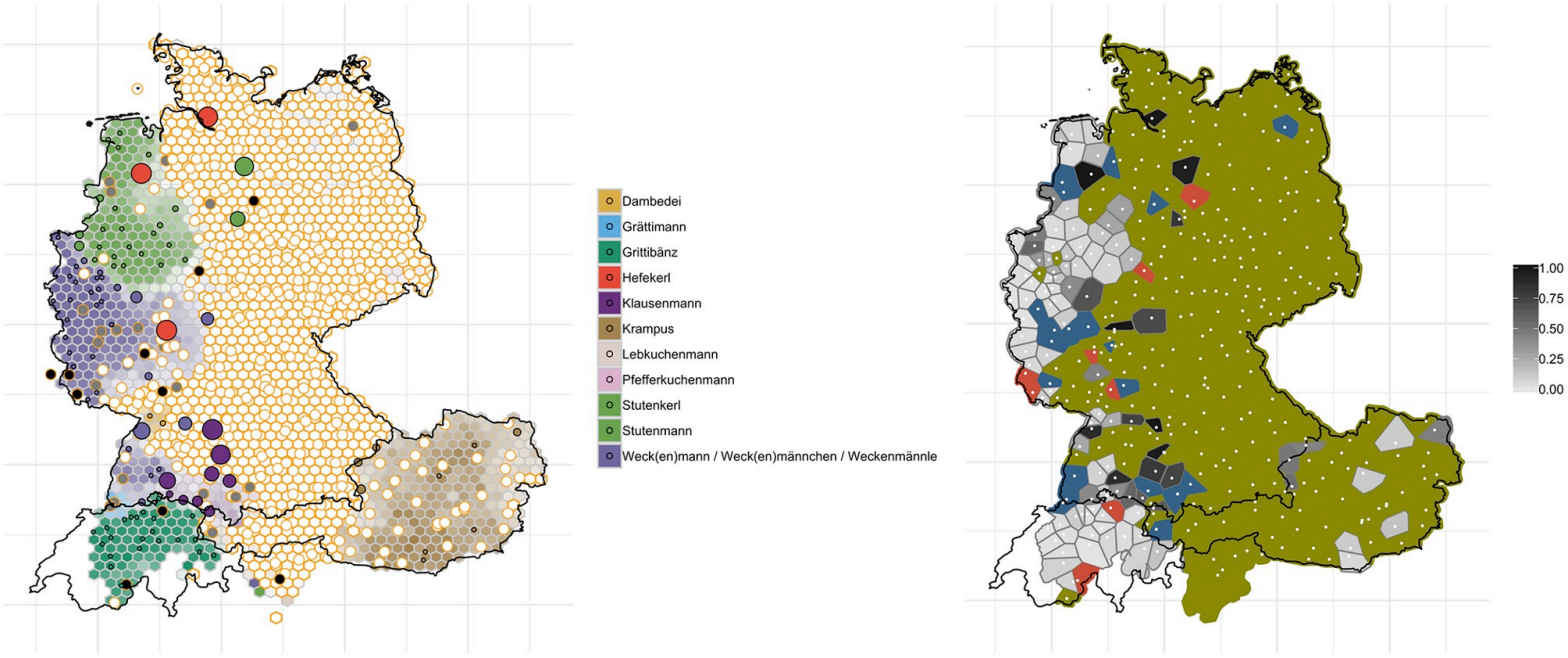
Variant and degree of change maps for ‘breadman’.

The changes we observe for ‘breadman’ are shown in Fig 7. Most interesting, here, is that many of the respondents did not have a word for this pastry–shown in white circles with orange outlines (contemporary data). The ‘word unknown’ responses in the west of Germany and in most of Austria may be due to the fact that there are regionally different types of preparation of this pastry and that the *WDU* specifically asked for a yeasty breadman with raisins which may not correspond to the traditional preparation in some regions.

While for much of the eastern part of Germany this pastry used to be unknown in *WDU* (white circles with orange outlines), today we find a substantial proportion of *Lebkuchenmann* (light brown) and *Pfefferkuchenmann* (pink) in these regions (see also *AdA* data from 2009/2010, which already shows substantial mentioning of *Lebkuchenmann* in Eastern Germany, round 7 question 1b). Here, again, the different traditions of preparing the breadman may be taken into account (*Lebkuchenmann* and *Pfefferkuchenmann* are made from gingerbread). In the southwest of Germany (north of Basel), however, we observe some trends of levelling: near Lake Constance we find that regionalized variants such as *Klausenmann* (dark purple) have become less known by the users in our contemporary data, as shown by the larger dots in that region–indicating that this variant is receding and being replaced by ‘word unknown’. The right-hand map in Fig 7 confirms these trends: very little change has occurred in German-speaking Switzerland and in western Germany. It has to be conceded, however, that some of the changes observed for the variants for ‘breadman’ may be attributed to a changed knowledge of the object or even to slightly altered concepts, due to changing traditions.

#### 3.1.2 Unstable center and north, stable south

**Fig 8:**
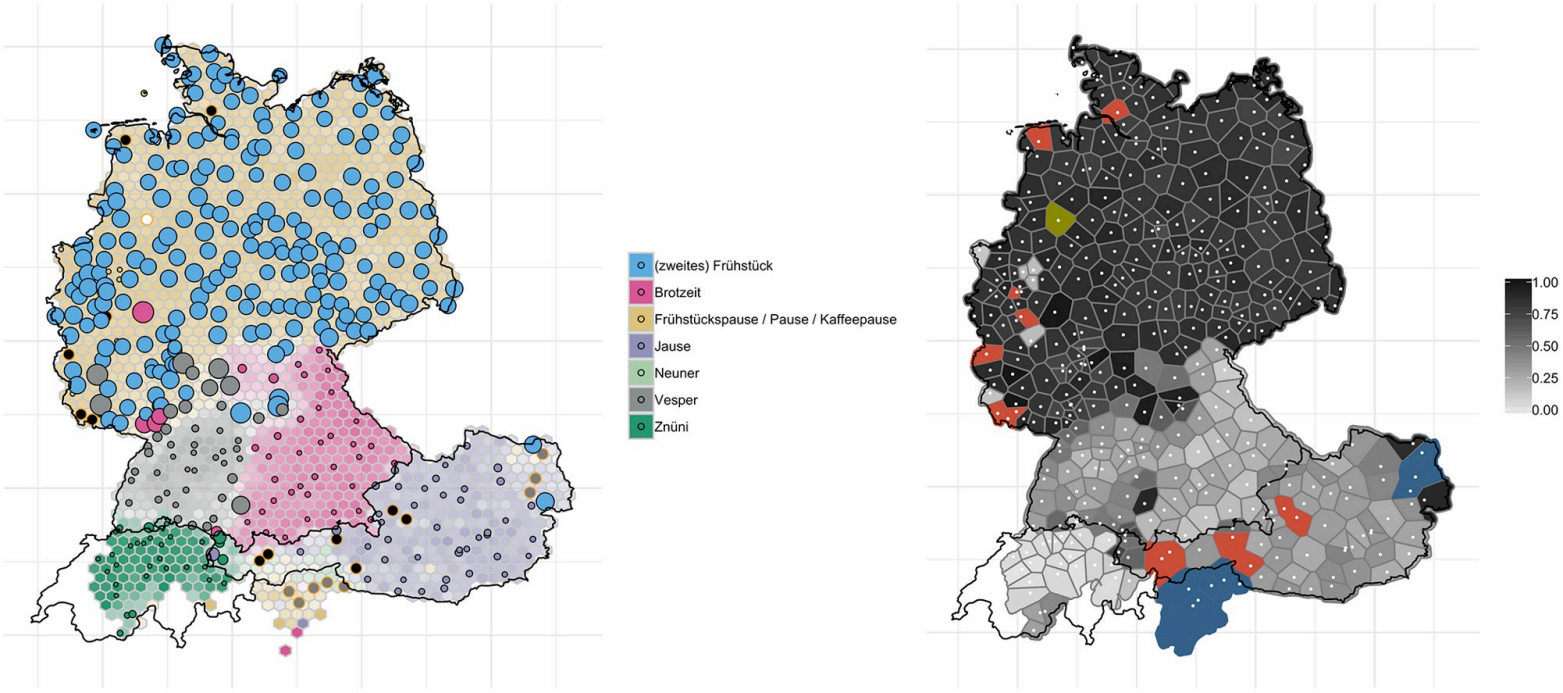
Variant and degree of change maps for ‘(second) breakfast that people have at their workplace’.

In the three exemplary variables introduced so far, it became evident that substantial regional levelling occurred for a number of variants. It further emerged that this regional levelling is particularly prevalent in the center and the north of Germany. In German-speaking Switzerland, on the other hand, little change has taken place, as seen for example in ‘breadman’. In this section, we explore further how regions differ in terms of degree of change that has taken place: we illustrate this by examining the variants for ‘(second) breakfast that people have at their workplace’ and ‘pencil case’. One variable that shows how regions differ in degrees of change is ‘(second) breakfast that people have at their workplace’, see Fig 8.

According to *WDU*, north of the river Main–an isogloss which German dialectologists refer to as *Speyerer Line*, separating Upper German in the south from Central German north of it [4,35]–speakers used to say *(zweites) Frühstück* (blue, left-hand panel) (i.e. ‘second breakfast’). Today, however, virtually all of these localities have changed to *Frühstückspause / Pause / Kaffeepause* today (light brown). There are some exceptions in Rhineland-Palatinate and Saarland, where *Vesper* (grey) used to be dominant, which is now being pushed aside by *Frühstückspause / Pause / Kaffeepause*. For the rest of German-speaking Europe, however, very little has changed: *Vesper*, the typical variant of Baden-Württemberg, the Bavarian *Brotzeit* (pink), the Swiss *Znüni* (dark green) and the Austrian *Jause* (purple) have remained largely unaffected by this change. On the right-hand side map in Fig 8 it is evident that much of the north of Germany is colored in dark grey, illustrating that more than 75% of the localities reported a different use from the *WDU*.

**Fig 9:**
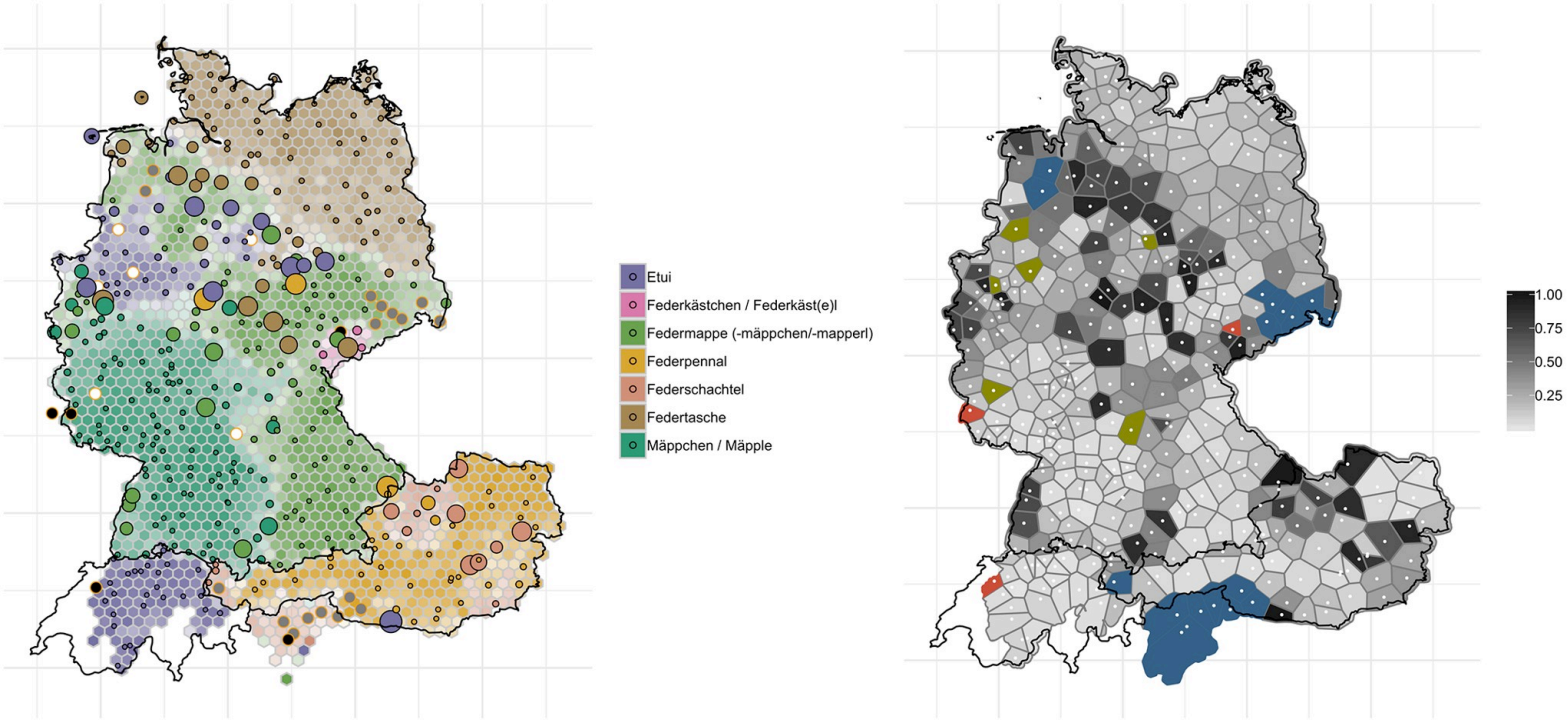
Variant and degree of change maps for ‘pencil case’.

The map for ‘pencil case’ shows similarly interesting patterns in terms how regions differ with regard to where change has taken place the most:

Fig 9 shows levelling for many regions in the north of Germany, but also in eastern Austria. In the northwest of Germany, variants such as *(Schul)-Etui* (purple) appear to be pushed aside by more widespread variants such as *Federmappe* (light green). Similarly, *Federtasche* (brown) is losing ground in Thüringen at the cost of *Federmappe*. In Lower Austria, *Federpennal* (dark yellow) appears to be gaining ground at the cost of the more localized *Federschachtel* (salmon). In Saxony, *Schiefermappe* and *Schieferkästl* were in use back at the time of the *WDU*, which we did not query. Similarly, for South Tyrol, *Griffelschachtel* was in use, which we also did not query (hence, these areas are colored in dark blue in the right-hand map in Fig 9). In German-speaking Switzerland and much of southern and central Germany, however, very little has changed in the past decades. The right-hand panel in Fig 9 confirms this pattern, albeit hot spots of change (i.e. dark spots) are much more scattered here compared to ‘(second) breakfast that people have at their workplace’ (cf. Fig 8). The Swiss German *(Schul)-Etui* (purple) and the central and southwestern German variant *Mäppchen/Mäpple* (turquois) are virtually unaffected by the change–as reflected in the bright grey polygons on the right-hand map in Fig 9. This lack of change in the south is already present when comparing the corresponding *AdA* map from 2006/2007 (round 4, question 12a) to our current data.

#### 3.1.3 Changes in East Germany and along the former German-German border

As mentioned in section 1, [19] compared data from *WDU* from the 1970s to the results of a pilot study for the *AdA* [12], with data collected in 2002. The study had indicated a divergence effect at the political border(s) between west and east Germany. Historically (until 1990), the present-day borders between the eastern German states Mecklenburg-Western Pomerania, Brandenburg, Saxony-Anhalt, Thuringia, Saxony and the west German states Schleswig-Holstein, Lower Saxony, Hesse and Bavaria correspond to the border between the former Federal Republic of Germany (FRG) and the German Democratic Republic (GDR). In two notable instances, the expressions for the temporal concepts ‘Saturday’ and ‘5:45’, this political border had more or less developed into an isogloss between western and eastern variants. *Sonnabend* (‘Saturday’) used to be distributed in the entire north and eastern parts of Germany. Some 25 years later, in the west this variant was largely replaced by *Samstag*, whereas *Sonnabend* was still the dominant variant in the east and so developed into one of the lexical shibboleths of the east of Germany. Similarly, the isogloss between *Viertel vor sechs* and *Dreiviertel sechs* (‘5:45’) had shifted eastwards so that it then corresponded to said political border (except for the southern border of Thuringia, Saxony and Bavaria, which also has *Dreiviertel sechs*, cf. http://www.atlas-alltagssprache.de/wp-content/uploads/2012/05/17_45.jpg).

**Fig 10:**
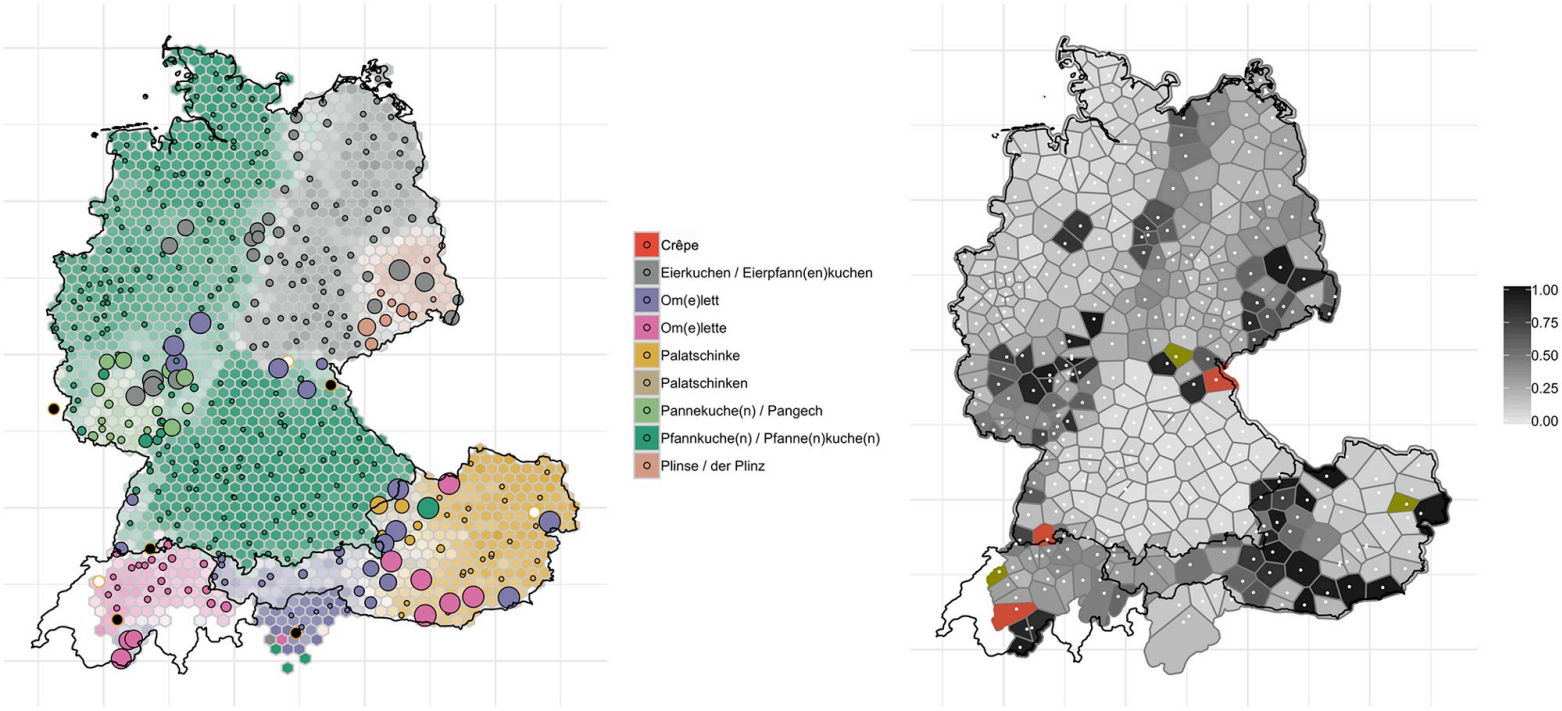
Variant and degree of change maps for ‘pancake’.

Similar real-time changes are observable in the results of a comparison of *WDU* and our contemporary data. Fig 10 shows the development of the variants for ‘pancake’. On the west German side of the border between the former FRG and the former GDR, *Pfannkuchen* (dark green, left-hand panel) has gained ground at the expense of *Eierkuchen* (grey) (a trend already observable in the corresponding *AdA* map from 2009/2010 (round 7, question 1a)). At the same time, the variant *Plinse* (salmon) has lost ground in Saxony and it appears to have been pushed back to its central area of distribution in the far-eastern bilingual Sorbian-German regions. As a consequence, both developments have resulted in a consolidation of *Eierkuchen* as the dominant east German variant.

**Fig 11:**
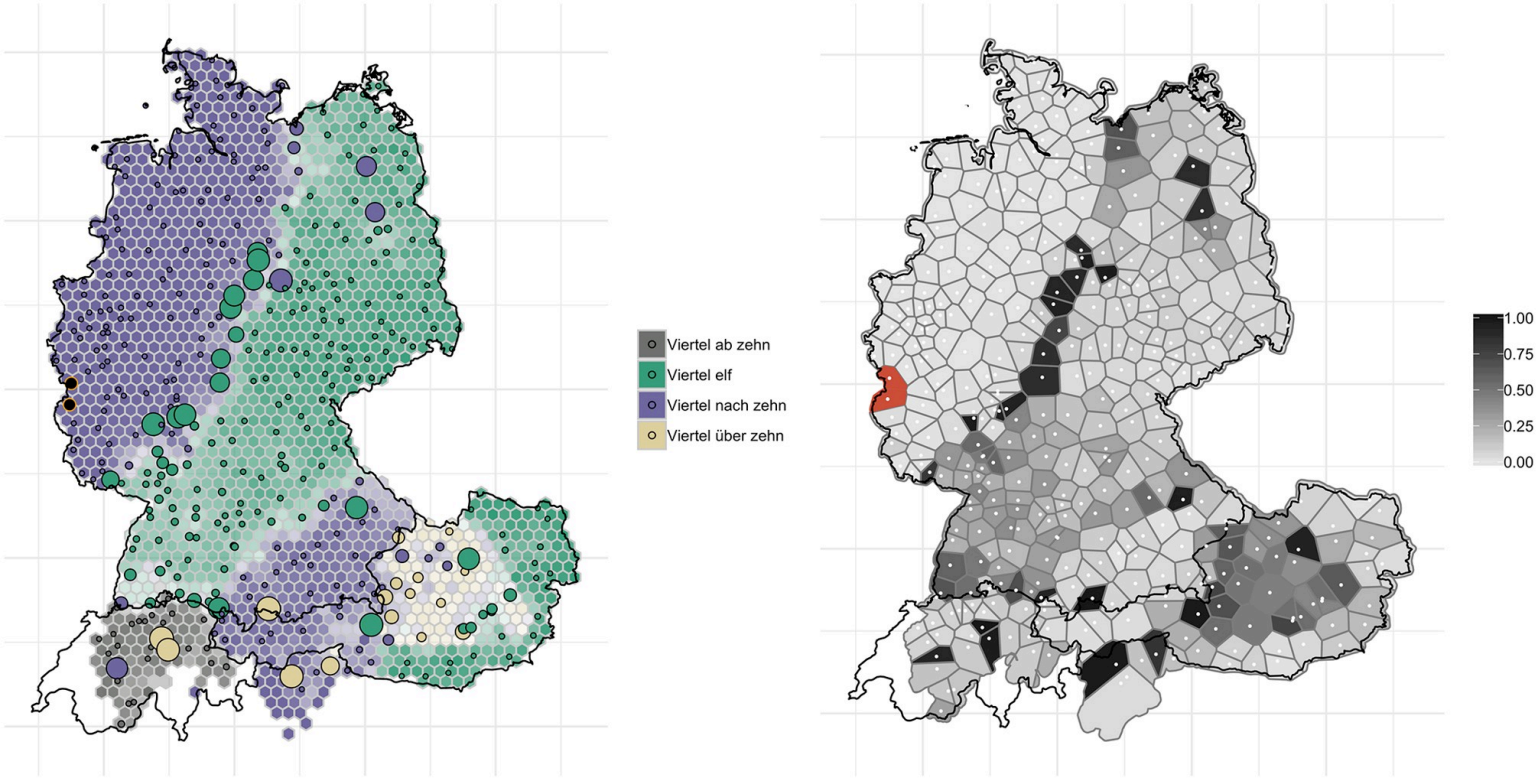
Variant and degree of change maps for ‘10:15’.

Fig 11 shows the variants for ‘10:15’ (the corresponding *WDU* map (vol. 1, map 40) elicited ‘6:15’). The regional distributions have not changed considerably over the past 40 years. However, we can observe a shift of the isogloss which runs across the northern and central parts of Germany and which separates the areas with *Viertel nach zehn* (purple, left-hand panel) and *Viertel elf* (turquois)–with the northwestern variant *Viertel nach zehn* gaining ground and pushing aside *Viertel elf* (the map on ‘10:15’ (round 7, question 11e) from *AdA* 2009/2010 already shows how *viertel nach zehn* is gaining ground in these regions).

These patterns of change are again reflected in the right-hand panel in Fig 11. There is a dark grey corridor following the isogloss between the *Viertel nach zehn* and *Viertel elf* areas, where substantial change appears to have occurred. A look at the corresponding map from the *AdA*, which features the political borders, reveals that a divergence has taken place right along the border between the former FRG and the former GDR by way of an ‘exchange’ of variants.

**Fig 12:**
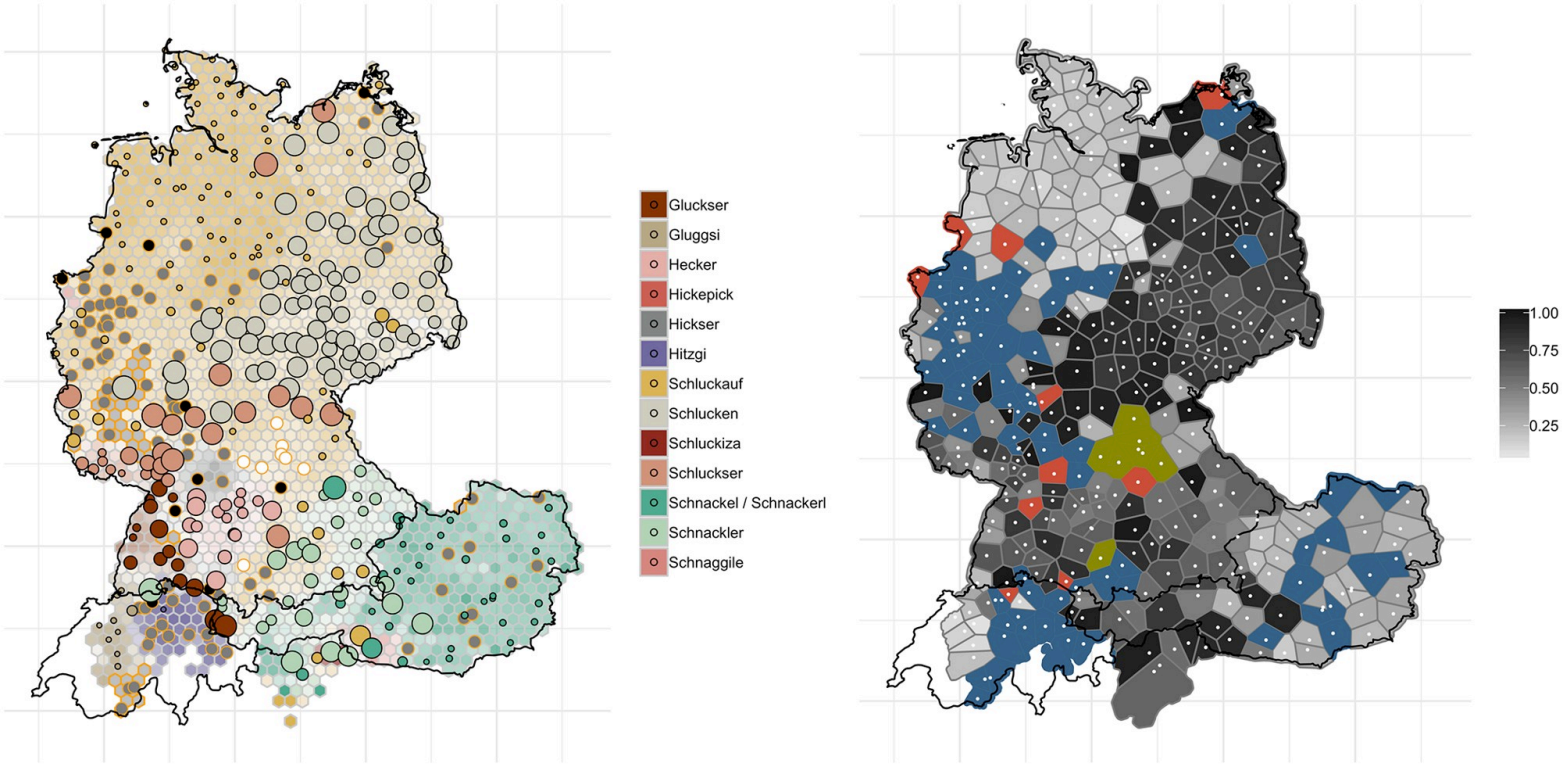
Variant and degree of change maps for ‘hiccups’.

Not in all cases, however, East German variants have been maintained. The most dramatic changes become apparent in the east of Germany when looking at the map for ‘hiccups’, cf. Fig 12. The panel on the left shows that the word *Schlucken* (light yellowish grey) has been largely abandoned in favor of the more supraregional (and indeed standard) variant *Schluckauf* (dark yellow) (in 2013, *AdA*, too, shows how *Schlucken* has largely been replaced by *Schluckauf* in these regions (round 10, question 3c)). The panel on the right reveals the extent of the change. Most of the polygons in the eastern part of Germany are dark grey or even black, indicating substantial change.

**Fig 13:**
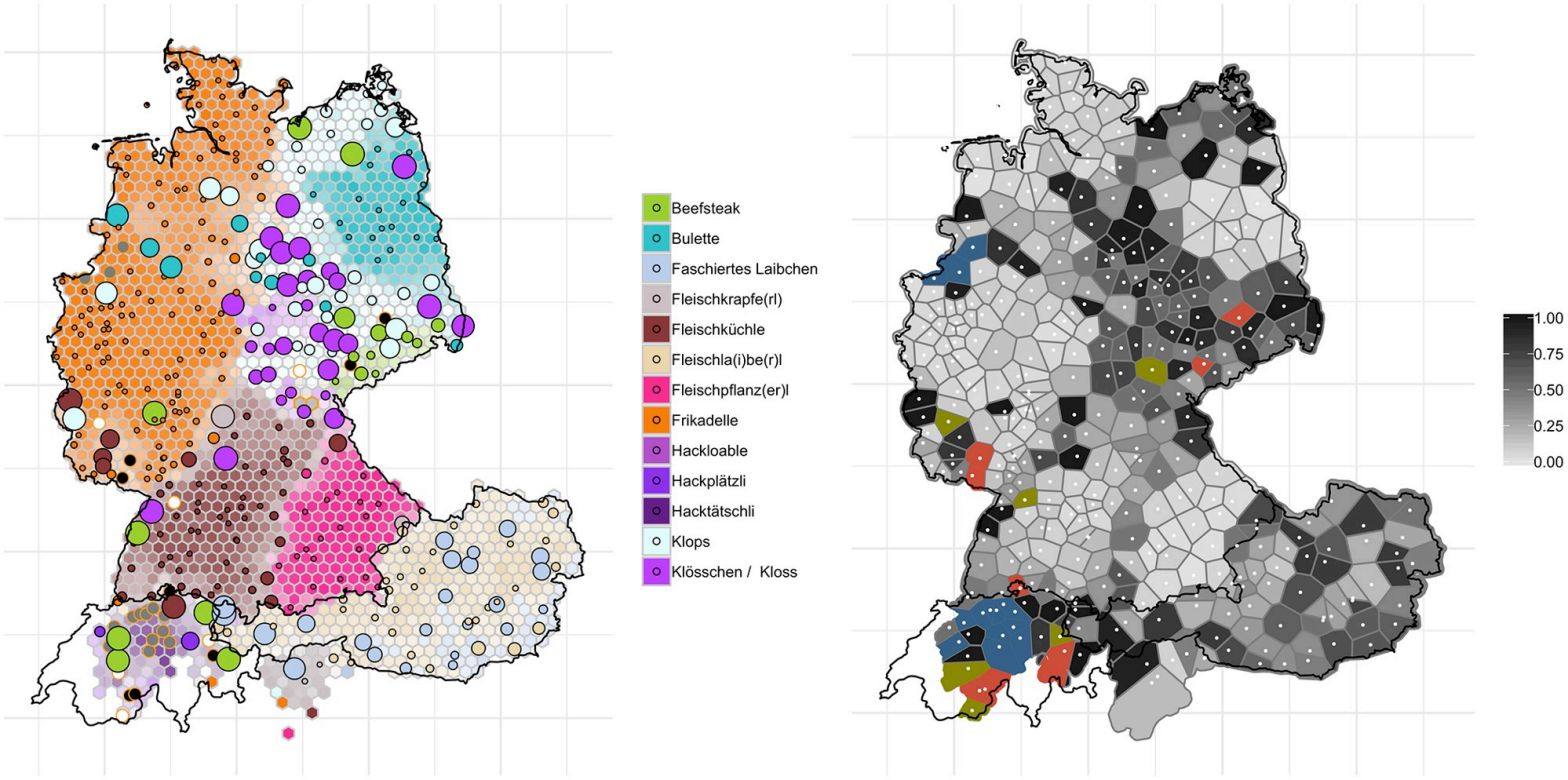
Variant and degree of change maps for ‘beef patty’.

Similarly, most changes on the map for ‘beef patty (made from ground beef, sometimes also pork)’, cf. Fig 13, appear to have occurred in the east of Germany. Particularly in Saxony-Anhalt and Thuringia, the use of *Kloß* (light purple) and its diminutive variant *Klößchen* (ibid.) seems to have been largely abandoned in favor of *Klops* (light blue). In Saxony and also in Mecklenburg-Western Pomerania, *Beefsteak* (green) has lost ground in favor of *Klops* or of *Bulette* (bright blue) (this same trend of *Beefsteak* losing ground to *Klops* is already observable in the *AdA* data from 2009/2010 (round 7, question 1f)). Again, the right-hand panel shows that most of the changes, to be recognized by the dark coloring of the hexagons, are concentrated in the east of Germany. Strikingly, these leveling processes are limited to the territory of the eastern German states. In the end, we are still dealing with variants that can be considered genuine east German shibboleths, i.e. *Klops* and *Bulette*.

#### 3.1.4 Aggregated change

**Fig 14:**
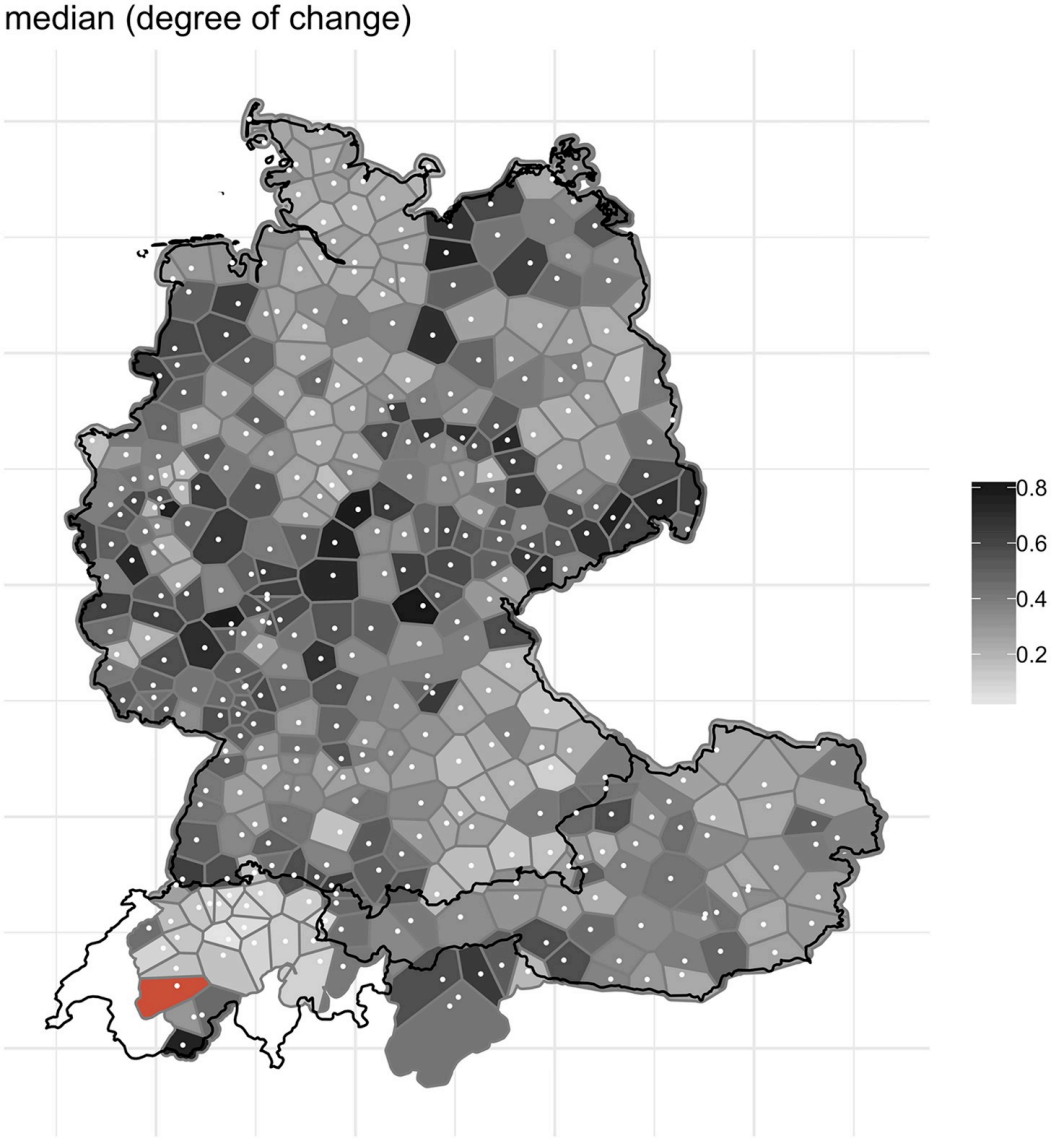
Median degree of change aggregated across 14 variables.

We can now aggregate these degrees of change across the 14 variables, cf. Fig 14.

Fig 14 reveals a relatively clear picture with regard to which regions changed the most (dark grey) and the least (light grey) over the past decades. The regions which are most affected by change are in the central west, the central east and the north east of Germany, but also in South Tyrol. Most of these regions of substantial change are north of the so-called *Main line*. This line, following for the most part the course of the river Main, has been identified as the most notable isogloss for lexis of colloquial German; it divides the northern and southern regions of German-speaking Europe considerably further south of the famous *Benrath line*, which in German dialectology serves as the traditional isogloss between Low German dialects in the north and High German dialects in the south [14,36,37]. Some parts of Austria, too, demonstrate substantial change across the 14 variables. Regions such as German-speaking Switzerland (especially central and eastern Switzerland), the northwest and the southeast of Germany (parts of Bavaria), on the other hand, appear to have undergone relatively little change in these 14 variables.

### 3.2 Change in apparent-time

**Fig 15:**
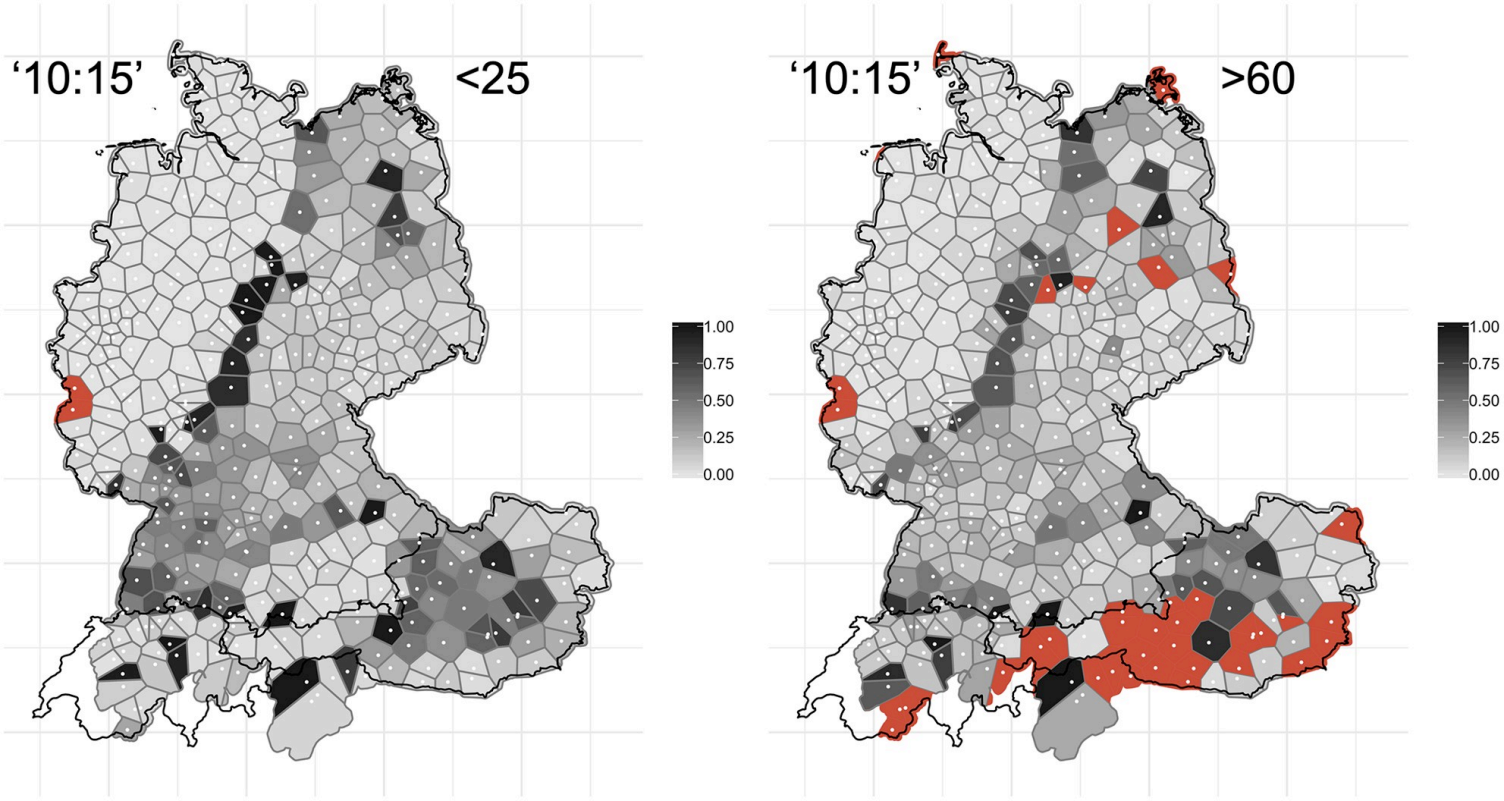
Degree of change maps for ‘10:15’. Users <25 years old are shown on the left, users >60 are shown on the right.

Section 3.1 provided a real-time analysis of language change, where the distribution of variants are compared at different moments in time (*WDU* vs. contemporary data). One way to test the validity of this real-time paradigm is to perform analyses in apparent-time–where, in our contemporary dataset, the degrees of change of different age groups are compared amongst each other [38]. To validate the real-time analyses, one would expect the older section of the population in our sample to exhibit less change when compared to *WDU*, while the younger users demonstrate more change. For this purpose, we subset the data set into users <25 years of age (N approx. 167,000) and >60 years of age (N approx. 42,000), i.e. with c. 35 years, i.e. one generation, between the cohorts. Overall, we found the younger cohorts to demonstrate more change compared to *WDU* than the older cohorts. By way of example, Fig 15 shows the degree of change maps for ‘10:15’ for the young cohort (left-hand side) and the old cohort (right-hand side).

**Fig 16:**
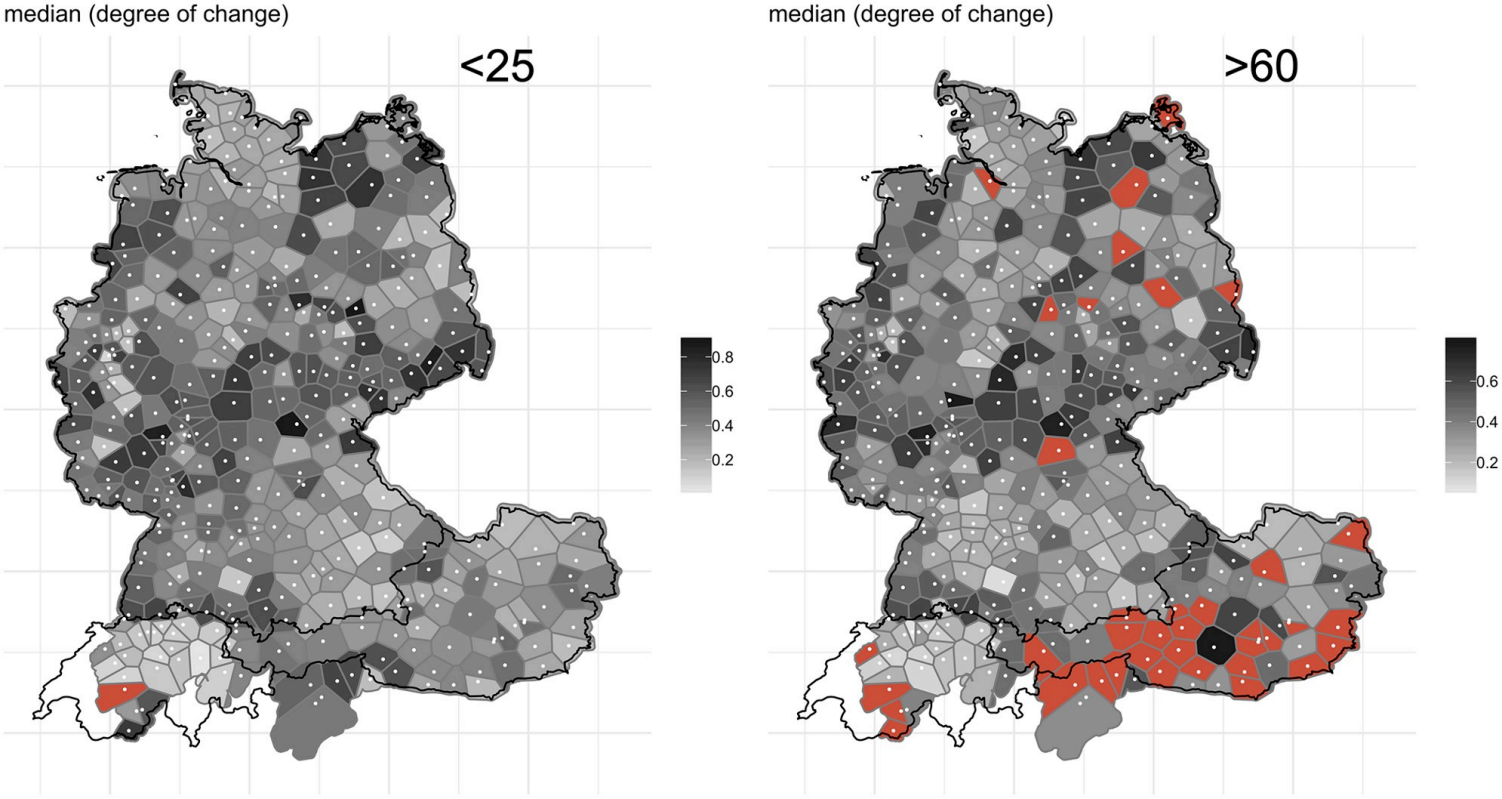
Aggregated change for users <25 years old (left) and users >60 years old (right).

Fig 15 reveals–overall–less obvious change for the older users, as shown by the lighter grey coloring of the map on the right-hand side. This is particularly evident in the corridor following the isogloss of *Viertel nach zehn* and *Viertel elf* variants, where change has been substantial. This corridor is considerably more distinct, i.e. darker, for the younger generation. Analogous to Fig 14, we aggregated the degrees of change values across the 14 variables for the two age brackets, see Fig 16.

Fig 16 perhaps shows–overall–darker polygons for the <25 cohort, which suggest more substantial change for the younger group when compared to the *WDU*. As a means of comparing the two age groups in relation to degree of change quantitatively, we performed a t-test to explore whether the difference between the two age groups in relation to degree of change was significant. <25 has a median of .39 (SD = .33), >60 a median of .37 (SD = .30). The t-test was significant (t = 3.049, p-value = .002).

## 4. Discussion

In this study we examined lexical variation in everyday language using web interactives as a new methodology of data collection. Going into the study we predicted that we would find substantial lexical change since the 1970s. In what follows, we want to explore potential reasons for this change in greater detail: 4.1.1 discusses reasons for levelling and 4.1.2. explores the role of political borders in the process of change. Finally, we discuss methodological limitations (4.2) and potential ways of validating this new paradigm of data collection (4.3).

### 4.1 Reasons for language change

#### 4.1.1 Leveling

In sections 3.1.1 and 3.1.2, we reported that variants with geographically broader usage are becoming more dominant at the cost of more localized, regional variants. As an example, this is the case for *Pantoffeln*, a both colloquial and standard variant for ‘slippers’ which is spreading and gaining ground at the expense of *Schluppen / Schluffen* and *Schlapfen*, which are all considered to be less standard than *Pantoffeln*. This phenomenon, typical of language change in progress, is often referred to as *levelling*. There are different reasons for regional levelling and for supralocal norms to emerge. In this context, a principle of frugality may play a role, where speakers move away from linguistic diversity as a means of being more frugal and at the same time wanting to become supralocally understood [39]. This principle can lead to a replacement of more small-scale variants by either wide-spread supraregional variants or standard forms (with or without a national distribution). One critical factor in lexical change, in this context, is increased geographical and social mobility of the speakers [40]:

1. Social mobility may lead to levelling as a result of an orientation towards standard varieties [41]. This, however, only applies to areas where the standard variety has gained high prestige in colloquial registers, such as in the central regions in Germany, whereas e.g. in Switzerland (and Liechtenstein) and large parts of Bavaria (and Austria) more local/dialectal varieties still enjoy a high (covert) prestige in everyday conversation. This may explain why in the latter regions the use of lexical variants has remained relatively stable and why these regions have not experienced such a considerable lexical change.
2. Geographical mobility is known to be a significant driver of language change [42,43]. It may lead to levelling as a result of contact between regional varieties. Contact between regional varieties, however, “does not necessarily lead to convergence towards the standard language” [44]; speakers may decide to give up more localized variants in favor of supralocally used variants which have high “indexical potential” [45] e.g. variants associated with major conurbations or with a regional identity.

The effect of these two sometimes conflicting processes can be seen on the map for ‘slippers’ (cf. Fig 6 above). Whereas in the central west region of Germany, speakers have replaced more localized variants such as *Schluffen* and *Schluppen* (at least the latter one is considered non-standard, cf. [46]) by *Pantoffeln*, a supraregional standard variant, in the northwest, the regional variant *Puschen* has become the dominant variant and has pushed aside not only the non-standard variant *Latschen*, but also the standard forms *Pantoffeln* and *Hausschuhe*. *Puschen* has developed into a popular regional marker in the north, not least because it is supported by the widespread idiom *in die Puschen kommen* (‘to get one’s act together’). Another example is the spread of the variant *Bulette* for ‘beef patty’ from the Berlin area to large parts of east of Germany, pushing aside more localized variants such as *Beefsteak*, (*Fleisch*-/*Gehacktes*-) *Kloß*/*Klößchen* and (*Brat*-) *Klops* (cf. Fig 13). The map for ‘(second) breakfast that people have at their workplace’ showed a high degree of levelling in north and central Germany where the word *Pause* (‘break’) and its relatively transparent compounds *Frühstückspause* (literally ‘breakfeast break’) and *Kaffeepause* (‘coffee break’), have become the dominant variants, whereas in most other areas, regionally salient variants (*Vesper*, *Brotzeit*, *Jause*, *Neuner*, *Znüni*), some of which are also quite opaque (e.g. *Vesper*, from Latin *vespera* ‘evening’, and *Jause*, from Slovenian *júžina* ‘lunch’), have remained relatively stable. There are also instances, however, where regional, relatively localized non-dominant variants have actually spread: Fig 7 revealed, for example, that *Weck(en)mann* (in the northwest of Germany) and *Krampus* (in the east of Austria) are extending into regions where previously respondents did not have a word for this variant. It has been observed elsewhere that the spread of regional lexical variants, in particular, can be attributed to the fact that they have become indices of regional identity and thus helped to “enregister” regional varieties, thus–to use the terminology of traditional sociolinguistics–turning from being associated with *covert prestige* to *overt prestige* [47]. While most of these studies focus on phonological variation, our study demonstrates the indexical potential of lexical variants. The overall trends of language change reported here through real-time analyses were corroborated by analyses of apparent-time change: older generation speakers (>60) overall exhibit less change than their younger (<25) peers.

#### 4.1.2 Divergence at political borders

What was also striking for a number of variables was the effect of national borders and political borders within nations on language use. Whereas the variants for ‘pencil case’ have seen considerable change within Germany, the formerly dominant variants in Switzerland (*(Schul-) Etui*) and Austria (*Federpennal*) seem to have been consolidated. Whereas *Federpennal* can be regarded as an Austriacism, *(Schul-) Etui* is not a ‘pure’ Helvetism, as it is also used in northwestern regions of Germany. South Tyrol, however, has seen considerable change. Here, *Federschachtel* has spread and has become the dominant variant. Again, it is not an exclusive variant of this region, as it is also used in some parts of Austria. Similarly, the maps for ‘slippers’, ‘beef patty’ and ‘(second) breakfast that people have at their workplace’ show a substantial degree of variation and change in Germany, and, in contrast, a spread and consolidation of variants within the national borders of German-speaking Switzerland, Austria, Liechtenstein and German-speaking Italy. Here, these variants have become dominant but not necessarily specific for the respective country or region (‘slippers’: Switzerland *Finken*, Austria: *Schlapfen* or *Patschen*; ‘beef patty’: Switzerland *Hacktätschli*, Austria: *Flaischla(i)berl*, South Tyrol: *Fleischkrapfen/-krapferl*; ‘(second) breakfast that people have at their workplace’: Switzerland *Znüni*, Austria: *Jause*). In some cases, a clear process of divergence along national borders has taken place. Certain variants which were attested in the *WDU* as being used in border regions of neighboring countries have virtually disappeared, e.g. the southwestern German variant *Fleischküchle* from Switzerland, the Austrian *Fleischla(i)ber(l)* (both ‘beef patty’) from South Tyrol, or Austrian *Geldbörse(rl)* (‘wallet’) from Bavaria. In some cases, processes of consolidation can be explained with the conventionalization of lexemes in administrative contexts, such as *Schul- (Etui)*, *Federpennal* or *Federschachtel* having become the ‘official’ word for ‘pencil case’ at school, or *Probe* (in the canton of Bern–as opposed to *Prüfung/Prüefig* in the rest of German-speaking Switzerland) and *Schulaufgabe* (in the German state of Bavaria–as opposed to *Klassenarbeit* in most other German counties) as the specific term for ‘school exam’.

The most striking processes of divergence, however, seem to have taken place at a *former* political border, i.e. the border between West and East Germany (or Federal Republic of Germany, FRG, and German Democratic Republic, GDR, as they existed until 1990; see Section 3.1.3). The maps for ‘pancake’, ‘10:15’ and ‘beef patty’ demonstrate, firstly, a consolidation of (north- and/or central-) western and (north-/central) eastern German variants, and secondly, a divergence of such variants along the former Iron Curtain in Germany: *Pfannkuchen* vs. *Eierkuchen*, *Viertel nach zehn* vs. *Viertel elf*, and *Frikadelle* vs. (*Brat-/Fleisch-) Klops* or *Bulette*. The latter variants may have been regarded as ‘national variants’ towards the end of the GDR; over the twenty-eight years after the fall of the Iron Curtain, some of them have essentially become regional markers of East-German identity.

### 4.2 Methodological considerations

Finally, we want to reflect the methods chosen. This section will be structured in three subsections: (i) we explore the benefits of this new paradigm of collecting data, we (ii) point to some of the limitations of this paradigm of data collection, and finally, (iii) we discuss a way of validating the method selected.

#### 4.2.1 Advantages of using web-interactives as a data collection tool

This new method of data collection for the purposes of studying language variation and change brings with it a number of key benefits: (a) a collection and analysis of datasets of unprecedented scale; (b) wide engagement with the public, thereby creating broad, public impact; and, (c) more generally, pushing methodological boundaries using technological advancements that interface with a traditional field such as dialectology.

As for (a), studies in dialectology typically examine speakers that have been carefully selected. Often, this speaker selection is time-consuming and ultimately results in a limited pool of participants. Collecting language data through web interactives sidesteps this problem–as the potential participant pool is virtually unlimited (which creates other problems, however, see next subsection). Which leads to (b): the web interactive allows researchers to reach millions of users. Such large-scale dissemination strategies are becoming more important in times of Research Excellence Framework (REF) and, most recently, Knowledge Exchange Framework (KEF, [48])–as outlined in [49, 50]. The web interactive engaged with the users and–at the same time–revealed to them that dialect variation is very much alive. Whether or not frameworks such as REF and KEF are cogent is a matter of a different debate. Yes, web-interactives such as the one presented here enable wide dissemination of research questions and findings–but is wide dissemination and impact really a necessary feat of undertaking scholarly research? To what degree do frameworks such as REF and KEF put additional pressures on researchers by dictating the type of research one should pursue? Can every field of research transfer knowledge and methods equally? As for (c), using the web to collect data on regional variation, too, is not new: the Harvard Dialect Survey [27] for American English (c. 47,463 users), [12] for German-speaking Europe (up to over 20,000 users), or the endeavors by [51,52] for Swiss German (c. 14,000 users), and–most recently–the surveys by [53] for French (c. 30,000 users) have shown that the web can be used successfully to collect data from a broader population.

While these endeavors obviously had a wide reach as well, to the best of our knowledge none of them had such a wide impact as our quiz for German-speaking Europe. It was successful because, for one, we applied the concept of gamification, i.e. users engage with a system and, in return, they get a reward–which in our case was the prediction of their regional origin. Gamification for use in linguistics is not new. In 2015, [54] showed that gamification strategies can be useful (and reliable) for linguistic annotations in tasks on morphosyntax. In the same year, [55] explored how gamification (such as in the form of Duolingo) can be used to promote risk taking in the foreign language learning classroom–risk taking being a crucial driving force behind increasing students’ performance in various language learning tasks. A special collection to be published in *Linguistics Vanguard* [56] is devoted to how smartphone apps, applying principles of gamification, can be used to collect linguistic data (in auditory, response, and visual form) in typologically diverse languages. Secondly, collaboration with strong media partners was, obviously, very crucial and fruitful. *SPIEGEL ONLINE* has roughly 20M unique users per month, *Tagesanzeiger* has roughly 1.3M unique users per month. This combination of gamification and collaboration with a strong media partner has–to our knowledge–not been done before. This collaboration brings up the question of how science and data-driven journalism are distinguished and what opportunities and pitfalls such a collaboration entail. [57] defines data-driven journalism or simply ‘data journalism’ as ‘essentially any activity that deals with data in conjunction with journalistic reporting and editing or towards journalistic ends’. In science, on the contrary, we typically follow the scientific method of postulating hypotheses, collecting data, analyzing data, and interpreting the patterns found–all the while maximizing potential reproducibility and sustaining review by peers. The former, thus, is more of a top-down approach–data is available, and the journalists look for patterns; while the scientific method follows a bottom up approach of hypothesis statement, collecting data, and testing assumptions. Furthermore, data-driven journalism does not necessarily follow the maxims of reproducibility and wanting to sustain peer review. Collaborations can nonetheless be fruitful–ways of visualizing data and patterns are, from the authors’ point of view, typically more nuanced and perhaps progressive in data-driven journalism than in (soft) sciences like linguistics. Journalists can benefit from academics by learning from their academic rigor and pursuit of making studies replicable, for instance.

#### 4.2.2 Limitations of using web-interactives as a data collection method

There are number of limitations to the data collection method applied in this study. The greatest problem (and at the same time the greatest strength) of the current paradigm is that anybody could (and still can) participate in the survey. There is no control over who participates in the web interactive. Consequently, this means that we do not know whether the users go through the quiz trollingly or sincerely and whether or not they fill in the metadata on gender, age, and evaluation of the result felicitously. Moreover, users may go through the quiz multiple times–although [58] suggests that, typically, repeated participation in web-based surveys is <3%.

Further noise is added to the dataset on a number of different levels: (a) We do not know if our speakers are first, second, or third language German speakers. Obviously, this may have a large effect on the nature of the data collected. (b) We have some users who are very mobile, geographically and socially, and we have users who exhibit opposite patterns. (c) Further, to evaluate the quiz result (see section 2.1), users placed a pin on a map. The precision of where this pin is placed exactly strongly depends on the zoom level the users selected–with a higher zoom level enabling a much more precise placement. (d) Furthermore, it is reasonable to assume that speakers–for nostalgic reasons–tend to respond rather ‘hypercorrectly’ and click variants which, for instance, they fondly remember from their childhood (cf. [59], which in return may affect the sample obtained. (a) and (b) could have been preempted, had we asked more metadata either at the end of the quiz (right before they obtain the result) or right at the start of the quiz. Here, we would have run the risk of participants opting out of the quiz, however, given that an average page visit typically lasts less than a minute [60, 61]. (e) Another major problem is the users’ self-reporting of dialect variants–an issue that we also encountered in our studies with smartphone apps [16]. As described in section 2.1, we asked users to self-report which variants they use; or should we say, which variant they *think* they use. Here we have to trust the users’ linguistic intuitions. [62] however, has convincingly shown that lay people’s intuition about non-standard linguistic usage can be erroneous. He asked speakers what they think they say when they hang up the phone, i.e. their phrase for saying ‘goodbye’. Aside from this introspection data, he also collected actual real-life data, i.e. how they say ‘goodbye’ on the phone in reality. It turned out that there was substantial discrepancy between introspection and actual observation. This needs to be kept in mind when discussing and interpreting our data–which is essentially solely based on self-reporting and introspection.

### 4.3 Validation of methodology

**Fig 17:**
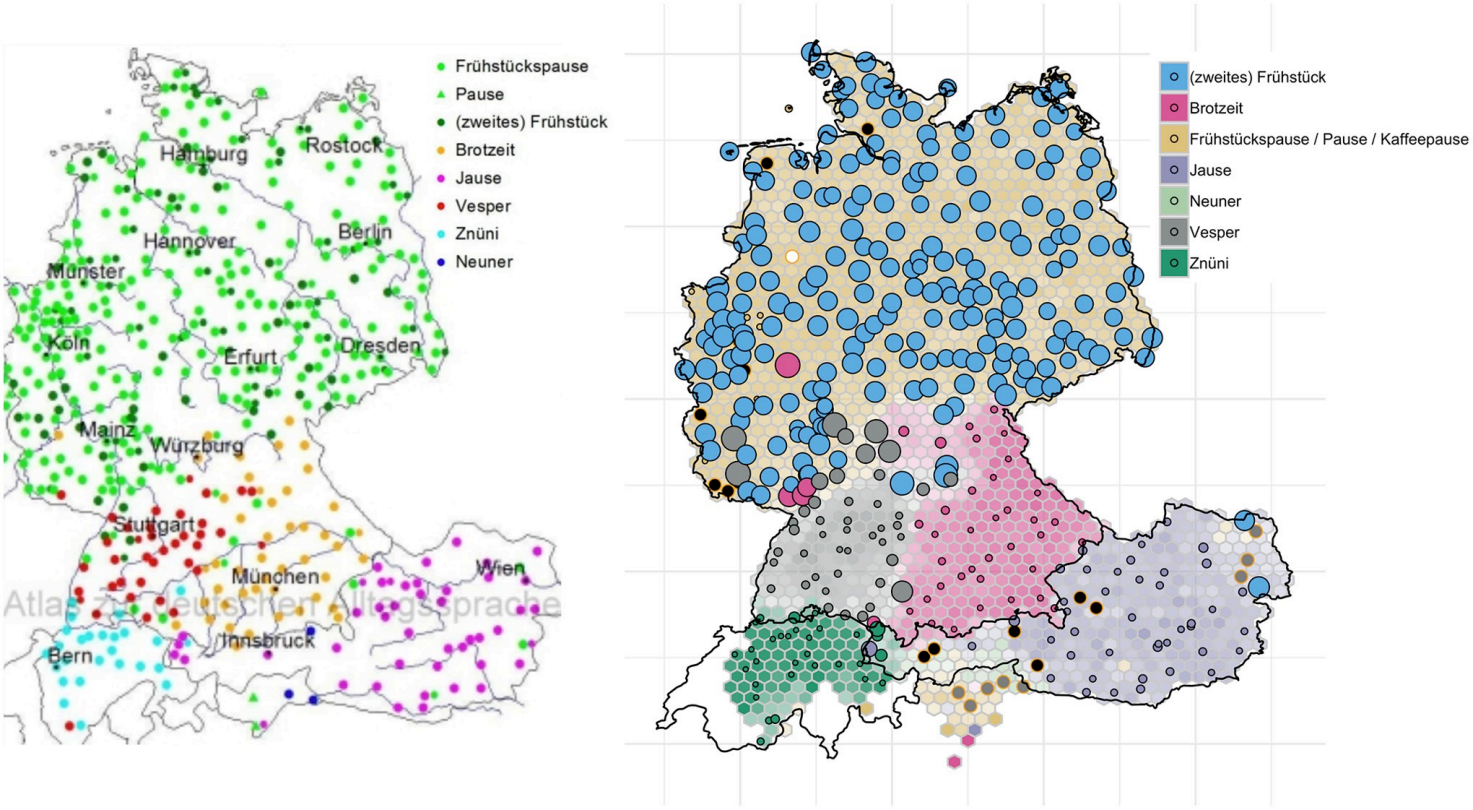
Regional distribution of variants for ‘(second) breakfast that people have at their workplace’. Right is our contemporary data (2015) and left is *AdA* (2003ff).

Given the plethora of methodological caveats mentioned in 4.2.2, the question of legitimacy of the current paradigm of data collection emerges. Are the findings and interpretations legitimate, given all the factors contributing to noise in the data presented in 4.2.2? Can we still make out the actual signal from the noise? One way to validate this is to explore, for example, whether Elspaß & Möller’s survey, *AdA*, shows similar results to our survey. In *AdA*, subjects were elicited in a much more controlled fashion than the participants in our study (cf. section 1). Fig 17 compares *AdA’s* survey round no. 4 question 2 from 2004/05, with c. 7.000 responses) result of ‘(second) breakfast that people have at their workplace’ (left) obtained through controlled elicitation and the same variable as collected through our web interactive (right).

Judging by the regional patterns evident in Fig 17 it seems as though we elicited highly similar patterns as *AdA* (2003ff.) did: users in German-speaking Switzerland reported *Znüni*, in much of Austria both surveys collected *Jause*, Baden-Württemberg shows *Vesper*, Bavaria *Brotzeit*, and north of the river Main, both surveys predominantly report *Frühstückspause*. What is interesting here, though, is that in the *AdA* data (left hand-side Fig 17), the historic *(zweites) Frühstück*–the same variant that was dominant in Eichhoff’s *WDU*–is still being used (dark green dots, left-hand panel) sporadically, whereas in our data, this has virtually disappeared from the map and change in this variant appears to have been concluded today; in *AdA*, this change appears to have been still ongoing. Given the very high degree of overlap in this and other variable(s)–in spite of the two different approaches to elicit data on regional variation–we argue that using web-interactives as a way of linguistic data collection can be very powerful and useful in the study of language variation and change.

## 5. Conclusions

This study set out to (a) determine the usefulness of a new crowdsourcing methodology for sociolinguistic research and to (b) examine linguistic change in German-speaking Europe with said methodology. This study has found substantial regional levelling particularly in the center and the north of Germany and has shown the effect of national and regional borders on lexical change. The scope of this study is limited in a number of ways, as addressed in 4.2.2. In spite of its limitations, the findings contribute to our understanding of regional variation and linguistic change in present-day German-speaking Europe; moreover, they underline the importance of updating surveys on colloquial speech periodically (and on lexis, in particular), as this has proven a highly dynamic area of our language. We presented an innovative methodology on how large data sets on regional variation can be collected. A natural progression of this work will be to apply this paradigm on other languages.
